# Coronavirus infectious bronchitis virus spike protein inhibits FUNDC1-mediated mitophagy to prevent nucleocapsid protein degradation

**DOI:** 10.1128/jvi.01800-25

**Published:** 2026-04-20

**Authors:** Jun Zhao, Jiaxin Tian, Liwei Zhang, Yingfei Li, Lihua Tang, Qila Sa, Ruotong Li, Jing Zhao, Ye Zhao, Guozhong Zhang

**Affiliations:** 1 State Key Laboratory of Veterinary Public Health and Safety, College of Veterinary Medicine, China Agricultural University 630101 , Beijing, China; 2 Key Laboratory of Animal Epidemiology of the Ministry of Agriculture, College of Veterinary Medicine, China Agricultural University 630101 , Beijing, China; University of North Carolina at Chapel Hill, Chapel Hill, North Carolina, USA

**Keywords:** IBV, mitophagy, spike protein, FUNDC1, host-virus

## Abstract

Autophagy is involved in various stages of the viral life cycle and modulates viral replication. Coronaviruses have developed several strategies to exploit autophagy for their benefit. Nevertheless, the exact mechanisms through which the infectious bronchitis virus (IBV) influences autophagy remain inadequately understood. Here, we demonstrate that IBV infection of chicken embryonic kidney (CEK) cells activates the AKT–mTOR signaling pathway to suppress autophagosome formation and mitophagy. Further investigation reveals that the viral spike protein (S) inhibits cellular autophagy by interacting with the mitophagy receptor FUNDC1. However, FUNDC1-mediated mitophagy promotes degradation of the viral nucleocapsid (N) protein and restricts IBV replication. To counteract this host defense mechanism, the S protein competitively binds to the LC3-interacting region (LIR) motif of FUNDC1, thereby disrupting its interaction with LC3 and ultimately suppressing mitophagy. Molecular docking analysis revealed that a conserved asparagine residue at position 240 (N240) in the S1 subunit of the IBV S protein is essential for binding to FUNDC1. Furthermore, reverse genetics demonstrated that an IBV mutant with an N240A substitution exhibited reduced pathogenicity in the kidneys, trachea, and lungs of specific-pathogen-free (SPF) chickens compared to the wild-type virus. Collectively, these findings unveil a novel mechanism by which IBV antagonizes host mitophagy and provide new insights into the host–virus interplay within the context of autophagic regulation.

**IMPORTANCE:**

IBV has evolved a mechanism to counteract the host’s antiviral defense. Specifically, the viral spike (S) protein blocks a form of autophagy called mitophagy by binding to the mitochondrial receptor FUNDC1. Normally, FUNDC1 helps cells eliminate damaged mitochondria and restricts IBV replication by promoting the degradation of the viral nucleocapsid protein. By interfering with this process, the S protein enhances viral survival. We further identified a single conserved amino acid in the S protein that is critical for this function, and mutation of this residue weakened IBV in chickens. These findings reveal how IBV manipulates host defenses and suggest new strategies for controlling coronavirus infections.

## INTRODUCTION

Infectious bronchitis virus (IBV), classified within the *Gammacoronavirus* genus, is a major pathogen in poultry, causing considerable morbidity and mortality with renal, respiratory, and reproductive complications (1–3). The ongoing genomic surveillance has identified numerous mutations in circulating viral strains, some of which may be linked to enhanced adaptation to mammalian cells, thereby raising concerns about potential cross-species transmission (4, 5). The IBV virus comprises four structural proteins: S, E, M, and N (6). The S protein is classified as a class I fusion protein and consists of two functional subunits: S1, which is responsible for receptor binding, and S2, which facilitates membrane fusion (7). Within the S1 subunit, both the N-terminal domain (NTD) and the C-terminal domain (CTD) are implicated in host receptor recognition (8).

Autophagy is a fundamental cellular degradation process, which can be categorized into selective and non-selective autophagy based on substrate specificity (9). Non-selective autophagy is activated under stress conditions, such as nutrient deprivation, leading to the random degradation of cytoplasmic components to provide energy and metabolic precursors. Conversely, selective autophagy specifically targets distinct cellular entities, including damaged organelles or protein aggregates (10, 11). A notable instance of selective autophagy is mitophagy, which eliminates dysfunctional mitochondria through pathways, such as the PINK1/Parkin ubiquitin-mediated cascade or receptor-mediated mechanisms involving BNIP3, NIX, and FUNDC1 (12). FUNDC1, a receptor located on the mitochondrial outer membrane, plays a crucial role in initiating mitophagy by binding to LC3 via a conserved LC3-interacting region (LIR) motif under hypoxic conditions, thereby maintaining mitochondrial homeostasis (13, 14). Recent studies have further highlighted the importance of FUNDC1 in preserving mitochondrial dynamics and its involvement in pathological conditions, including cancer, cardiovascular diseases, and metabolic syndrome (15). However, the association between FUNDC1 and viral interactions remains unexplored.

Viruses have evolved multiple strategies to manipulate autophagy (16). Multiple viruses regulate mitochondrial dynamics, mitophagy, and mitochondria-derived vesicles to promote their own replication (17–19). Recent studies have indicated that the main protease NSP5 of PDCoV cleaves the selective autophagy receptor NBR1, consequently inhibiting NBR1-mediated autophagic degradation of viral proteins, which, in turn, enhances viral stability (20). Additionally, the SARS-CoV-2 S protein has been shown to interact with the mitochondrial protein MAO-B in neuronal cells, enhancing MAO-B activity and inhibiting mitophagy, thereby contributing to the development of neurodegenerative diseases (21, 22). As early as 2013, research demonstrated that IBV suppresses autophagy signaling in CEK cells and avian DF1 cells (23), and that the coronavirus NSP6 protein limits autophagosome expansion (24). Nevertheless, the mechanisms by which other IBV viral proteins modulate autophagy, particularly the regulation mediated by the S protein, remain largely unexplored.

The virus replicates not only in the epithelium of the respiratory tract but also in multiple extra-respiratory tissues along the digestive tract and elsewhere, including the kidney, oviduct, and testes. Notably, currently predominant GI-19 (QX-like) strains are frequently associated with pronounced renal tropism and nephron pathogenicity (4, 25). Although it has been well-known that the kidney is a target organ for IBV infection, which leads to severe kidney damage and higher mortality, mechanistic investigations into its pathogenicity in kidney cells remain limited (26, 27). This gap is largely attributed to the technical challenges of establishing stable and reproducible avian infection models, which have impeded our comprehension of virus–host interactions within a physiologically relevant context. In this study, using primary CEK cells as a physiologically relevant model, we demonstrate that the S protein of IBV inhibits mitophagy by interacting with FUNDC1. Notably, the autophagy receptor FUNDC1 restricts IBV replication by facilitating the degradation of the viral N protein; the S protein binds to the LIR motif of FUNDC1, thereby impairing its autophagic function and abrogating its ability to degrade N. Furthermore, a mutation at a conserved residue in the S protein (Asn240) abolishes its interaction with FUNDC1 and markedly attenuates viral pathogenicity. Together, our findings uncover a previously unrecognized mechanism in which FUNDC1-mediated selective autophagy functions as a host antiviral defense, while revealing an evolutionary countermeasure by which IBV subverts this defense.

## RESULTS

### IBV infection inhibits mitochondrial autophagy *in vivo* and *in vitro*

**Fig 1:**
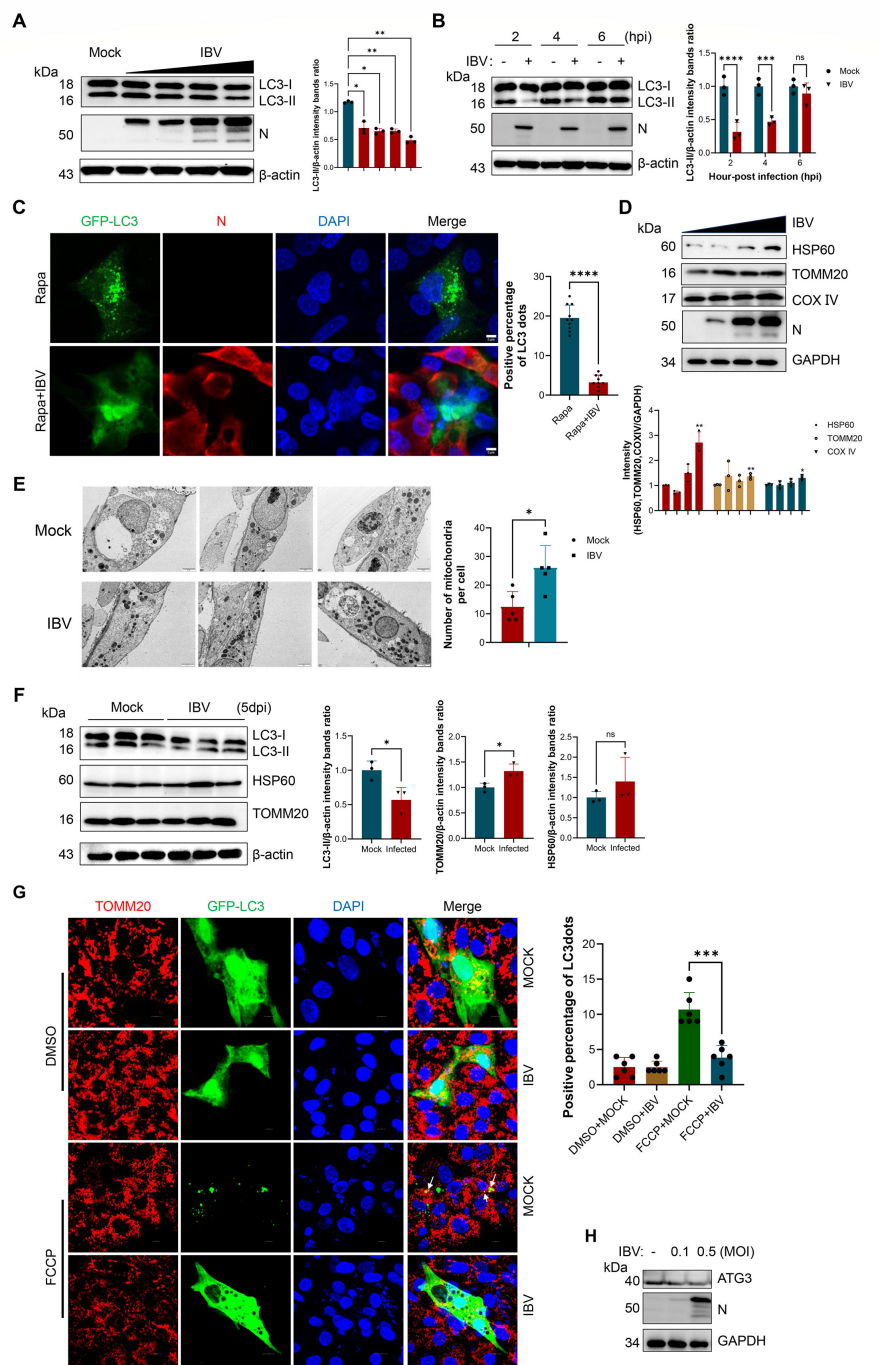
IBV infection inhibits mitochondrial autophagy. (**A**) CEK cells were infected with IBV at the initial concentration of MOI = 0.01 for 12 h. (**B**) CEK cells were infected with IBV at the MOI of 0.01 for 2, 4, and 6 h; cell lysates were then harvested to detect LC3-II transformation using western blotting. (**C**) Visualization of autophagic puncta via laser confocal microscopy. CEK cells were transfected with a plasmid expressing GFP-LC3 for 24 h and were then mock infected or infected with IBV at a MOI of 0.01 for another 24 h. The autophagic puncta were observed with laser confocal microscopy, and the positive percentage of cells that formed autophagic puncta was statistically analyzed. (**D**) CEK cells were infected with IBV at the initial concentration of MOI = 0.01 for 24 h. Then, the cell lysates were collected and detected using western blotting with the indicated antibodies. (**E**) CEK cells were mock infected or infected with IBV at a MOI of 0.01 for 24 h; the ultrastructure of autophagosomes was then observed using transmission electron microscopy. (**F**) One-day-old SPF chicks were infected with EID_50_ = 10^5^ (*n* = 3), and a mock infection group was set up. Kidney tissues were collected 5 dpi. Organized lysates were then harvested to detect LC3-II transformation, HSP60, and TOMM20 using western blotting. (**G**) CEK cells were transfected with GFP-LC3 for 24 h and were then mock infected or infected with IBV at a MOI of 0.01 for another 24 h in the presence or absence of FCCP (2.5 μM). The mitochondria were labeled using TOMM20. The colocalization of autophagosomes and mitochondria was visualized using laser confocal microscopy. (**H**) CEK cells were infected with IBV at different multiplicities of infection (MOIs) for 24 h, and ATG3 expression was analyzed by western blot; GAPDH served as the loading control. Data are mean ± SD from three independent experiments. Differences were considered significant at (∗) *P* < 0.05, (∗∗) 0.001 < *P* < 0.01, (∗∗∗) *P* < 0.001; ns, not significant*.*

To investigate the effects of IBV infection on autophagy, we developed an *in vitro* infection model using CEK cells. The CEK cells were infected with IBV at varying multiplicities of infection (MOI), and successful viral replication was verified through the detection of the N protein. Western blot analysis indicated a dose-dependent reduction in LC3-II accumulation with increasing MOI (Fig. 1A), suggesting an impairment in autophagosome formation. Considering that the IBV replication cycle is approximately 6 h, we subsequently investigated the temporal dynamics of autophagy inhibition (28). Notably, LC3-II levels were markedly reduced as early as 2 to 4 h post-infection (hpi) (Fig. 1B), implying that autophagy suppression occurs during the initial phase of viral replication. To further visualize autophagosome formation, CEK cells were transfected with GFP-LC3. In control cells treated with rapamycin (10 μM), numerous GFP-LC3 puncta were observed, indicative of robust autophagosome assembly, whereas IBV-infected cells exhibited diffuse cytoplasmic GFP-LC3 fluorescence (Fig. 1C). These findings demonstrate that IBV infection inhibits autophagosome formation in CEK cells.

In our subsequent investigation, we evaluated the effects of IBV on mitochondrial homeostasis. Immunoblotting analyses showed a dose-dependent accumulation manner of mitochondrial marker proteins, such as HSP60, TOMM20, and COX IV, following infection (Fig. 1D). The results from the transmission electron microscope also confirmed these findings. It showed that the number of mitochondria in the infected CEK cells had increased, and the mitochondria were damaged (Fig. 1E; Fig. S1A). To ascertain whether these effects manifest *in vivo*, one-day-old specific-pathogen-free (SPF) chickens were infected with IBV. At 5 days post-infection (dpi), kidney tissues were collected, and the infection was confirmed (Fig. S1B). Compared to uninfected controls, there was a significant reduction in LC3-II levels, alongside an elevation in TOMM20 levels (Fig. 1F), suggesting mitochondrial accumulation due to impaired mitophagy. Next, we examined the colocalization between GFP-LC3 puncta and TOMM20 by confocal microscopy. In the DMSO-treated groups, neither mock-infected nor IBV-infected cells displayed GFP-LC3 puncta, consistent with our previous observations. In contrast, FCCP treatment induced prominent GFP-LC3 puncta that colocalized with TOMM20, indicating the activation of mitophagy. However, IBV infection markedly disrupted this pattern, resulting in a diffuse distribution of GFP-LC3, further supporting the inhibition of mitochondrial autophagy (Fig. 1G).

Our previous work showed that IBV downregulates ATG3 (Fig. 1H), a critical component in the autophagosome elongation. To investigate whether the mitochondrial accumulation was attributable to the autophagy inhibition, we assessed mitochondrial protein levels in cells co-treated with IBV and ATG3. Overexpression of ATG3 partially restored mitochondrial clearance, as evidenced by the decreased levels of HSP60, TOMM20, and COX IV (Fig. S1C). Collectively, these findings indicate that IBV impedes LC3 lipidation and autophagosome formation both *in vitro* and *in vivo*, thereby inhibiting mitophagy and promoting the accumulation of damaged mitochondria.

### IBV suppresses autophagosome formation via the AKT–mTOR signaling pathway

**Fig 2:**
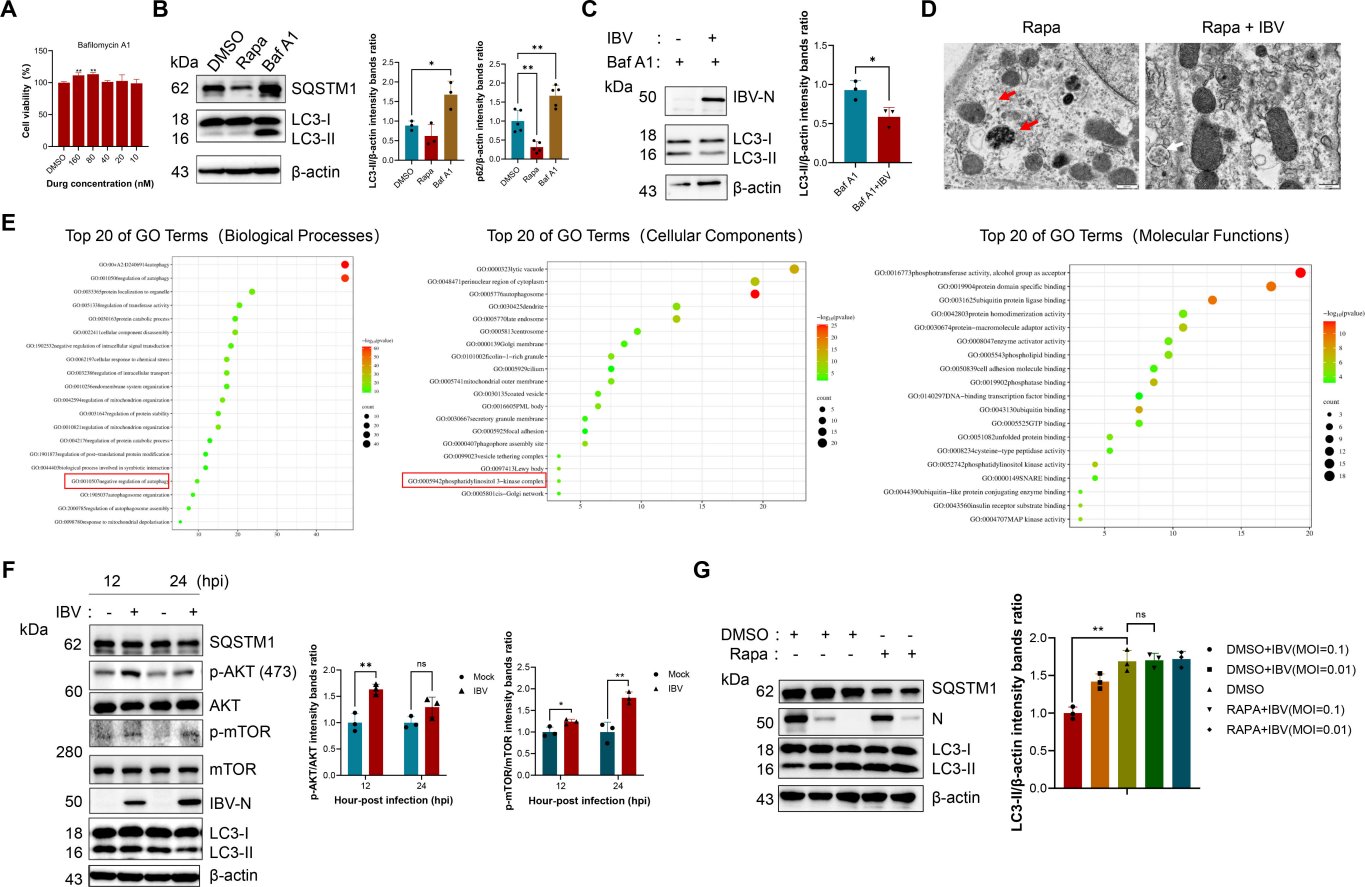
IBV suppresses autophagosome formation via the AKT–mTOR signaling pathway. (**A**) CEK cells were treated with different concentrations of Bafilomycin A1 (40 nM) for 6 h, and then, the cell viability was detected by the CCK-8 kit. (**B**) CEK cells were cultured in medium with the indicated DMSO, Bafilomycin A1, or rapamycin for 6 h and 24 h, respectively; cell lysates were then harvested to detect LC3-II transformation and SQSTM1 using western blotting. (**C**) CEK cells were treated with Bafilomycin A1 for 6 h and were then mock infected or infected with IBV for another 24 h at a MOI of 0.01. The cell lysates were analyzed by western blotting. (**D**) CEK cells were treated with rapamycin for 24 h and were then mock infected or infected with IBV for another 24 h at a MOI of 0.1; the ultrastructure of autophagosomes was then observed using transmission electron microscopy. (**E**) The top 20 enriched GO terms for biological process, cellular component, and molecular function. Rich factor refers to the ratio of the number of genes with the indicated term entry with respect to the total number of genes in that term. The size of the bubble indicates the number of genes, and the color of the bubble indicates the level of significance. (**F**) CEK cells were infected with IBV at the MOI of 0.01 for 12 and 24 h. Total protein was subjected to western blotting analysis using antibodies recognizing p-AKT, total AKT, p-mTOR, total mTOR, and β-actin as a loading control. (**G**) CEK cells were treated with DMSO or rapamycin for 24 h and then infected with different doses of IBV for 24 h, respectively. Cell lysates were then harvested to detect LC3-II transformation using western blotting. Data are mean ± SD from three independent experiments. Differences were considered significant at (∗) *P* < 0.05, (∗∗) 0.001 < *P* < 0.01, (∗∗∗) *P* < 0.001; ns, not significant*.*

Decreased levels of LC3-II may result from either impaired autophagosome formation or enhanced autophagic degradation. To investigate whether IBV suppresses autophagosome formation, we employed Bafilomycin A1 (Baf A1), an established inhibitor of autophagosome–lysosome fusion and autophagic flux (29). Treatment with 40 nM Baf A1 for 6 h did not compromise cell viability (Fig. 2A). Under these conditions, there was a significant increase in LC3-II accumulation compared to the DMSO control, indicating an effective blockade of autophagic flux. Similarly, levels of SQSTM1/p62 were elevated following Baf A1 treatment (Fig. 2B). These results confirmed that 40 nM Baf A1 for 6 h efficiently blocks autophagic flux in CEK cells. Next, CEK cells were pretreated with Baf A1 and subsequently infected with IBV. Strikingly, LC3-II accumulation remained markedly suppressed in IBV-infected cells (Fig. 2C), suggesting that IBV interferes with autophagosome formation at an early stage of the autophagy pathway. Consistent with these observations, transmission electron microscopy identified a substantial presence of double-membrane autophagosomes enclosing damaged organelles in the rapamycin-treated group (red arrows), whereas such structures were rarely observed in cells infected with IBV. Instead, double-membrane vesicles containing viral particles were occasionally observed (white arrows) (Fig. 2D).

We conducted a transcriptome analysis on the CEK cells 24 h after infection. Gene ontology (GO) enrichment analysis of differentially expressed genes highlighted significant enrichment in terms related to negative regulation of mitophagy and PI3K complex assembly (Fig. 2E). The above results indicate that IBV infection inhibits the generation of autophagosomes in CEK cells. Recent studies have reported that porcine epidemic diarrhea virus (PEDV, which belongs to the Alphacoronavirus) infection activates the AKT–mTOR signaling pathway (24). Guided by these results, we investigated whether IBV modulates the AKT–mTOR signaling pathway. Immunoblotting results demonstrated that phosphorylation of both AKT and mTOR was significantly elevated at 12 and 24 hpi compared to mock-infected controls (Fig. 2F), indicating activation of the AKT–mTOR axis by IBV. To test whether this pathway is responsible for IBV-induced autophagy inhibition, we treated infected cells with rapamycin, a specific mTOR inhibitor. Treatment with rapamycin (2 μM) effectively restored LC3-II accumulation in IBV-infected CEK cells (Fig. 2G). Collectively, these findings indicate that IBV suppresses autophagosome formation in CEK cells predominantly through activation of the AKT–mTOR signaling pathway. Although activation of the AKT–mTOR axis explains the general suppression of autophagosome formation during IBV infection, mitophagy can also be independently regulated through mitochondrial receptors. Given the pronounced accumulation of damaged mitochondria observed in IBV-infected cells, we hypothesized that IBV may additionally employ a more selective mechanism to directly target mitophagy.

### The IBV S protein inhibits mitophagy by interacting with the mitochondrial receptor FUNDC1

**Fig 3:**
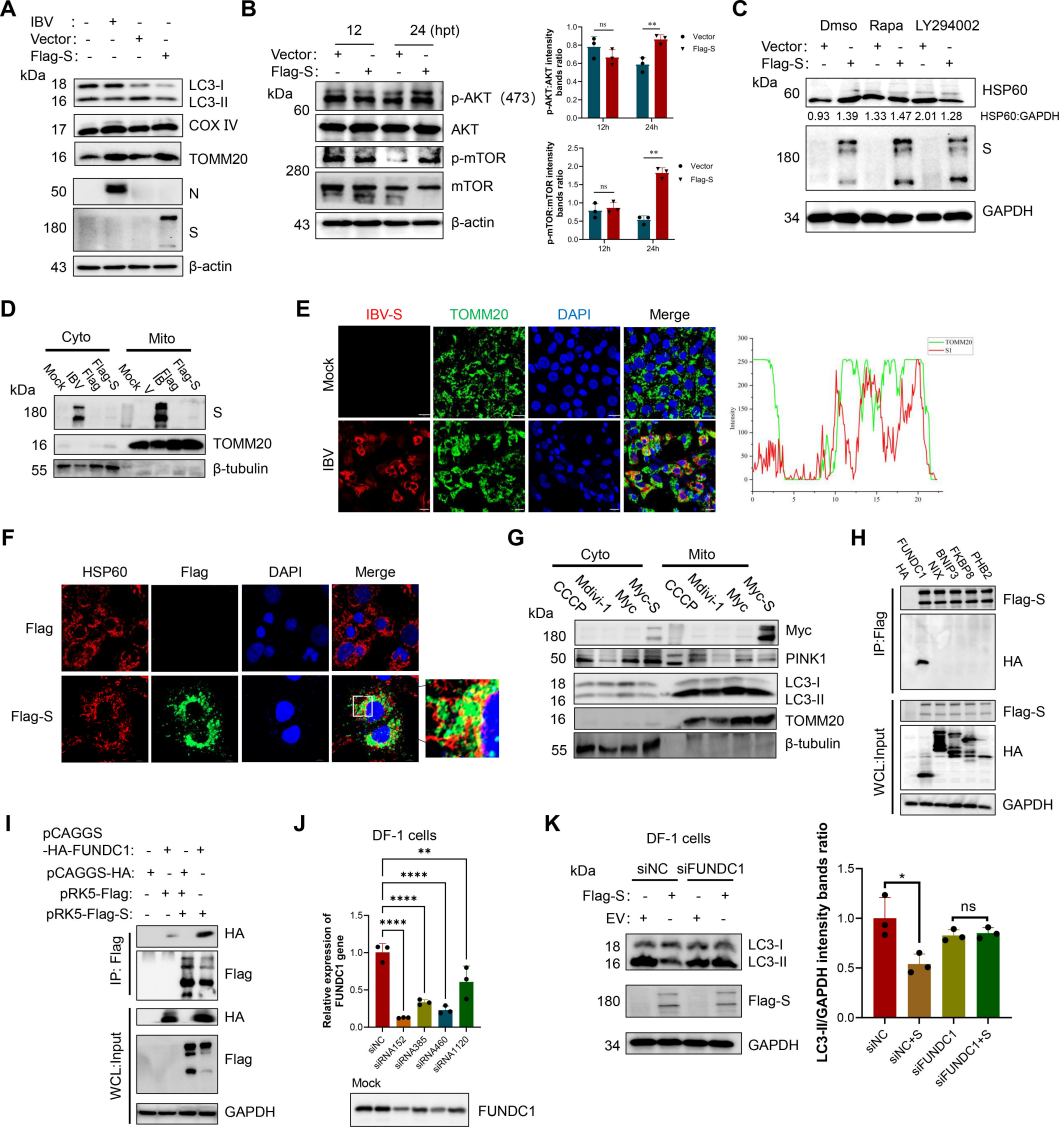
IBV S suppresses mitophagy via FUNDC1. (**A**) CEK cells were infected with IBV or transfected with pRK5-Flag-S plasmid for 24 h; cell lysates were then harvested to detect LC3-II transformation and the protein levels of TOMM20 and COX IV using western blotting. (**B**) The treatment of CEK cells was similar to panel **A**. Total protein was subjected to western blotting analysis using antibodies recognizing p-AKT, total AKT, p-mTOR, total mTOR, and β-actin as a loading control. (**C**) CEK cells were transfected with pRK5-Flag-S or empty vector for 12 h and then cultured in medium with the indicated DMSO, LY294002 (10 μM), or rapamycin (2 μM) for 24 h. The expression of HSP60 was detected by western blotting. (**D**) CEK cells were transfected with Flag-S or infected with IBV for 24 h. The cytoplasm without mitochondria and mitochondrial components was purified for western blotting analysis (TOMM20, mitochondria; β-tubulin, cytoplasm). (**E and F**) The localization of Spike protein and mitochondria was detected by confocal microscopy (TOMM20 or HSP60, mitochondria). (**G**) CEK cells were transfected with Myc-S or empty vector for 12 h or treated with the indicated CCCP (2 μM) or Mdivi-1 (5 μM) for 12 h. The cytoplasm without mitochondria and mitochondrial components was purified for western blotting analysis. (**H, I**) CEK cells were transfected with vector and Flag-S or HA-FUNDC1, HA-NIX, and HA-BNIP3, respectively. Cell lysates were subjected to IP. (**J**) DF-1 cells were transfected with FUNDC1-targeting siRNA or negative control (NC) for 24 h, and the expression of FUNDC1 was assessed by qPCR and western blotting. (**K**) DF-1 cells were transfected with siRNA for 24 h before Flag-S transfection, and the expression of LC3-II was detected by western blotting. Data are mean ± SD from three independent experiments. Differences were considered significant at (∗) *P* < 0.05, (∗∗) 0.001 < *P* < 0.01, (∗∗∗) *P* < 0.001; ns, not significant.

IBV infection has been shown to be associated with autophagy dysregulation in tracheal epithelial cells, and the SARS-CoV-2 S protein has been reported to suppress mitophagy and alter mitochondrial homeostasis (21, 30). Based on these observations, we examined the effect of IBV S protein on autophagy in CEK cells. Overexpression of the S protein resulted in elevated levels of the mitochondrial outer and inner membrane markers TOMM20 and COX IV, along with reduced LC3-II accumulation (Fig. 3A), indicating impaired autophagosome biogenesis. Furthermore, expression of the S protein for 24 h resulted in enhanced phosphorylation of AKT and mTOR (Fig. 3B). The pharmacological inhibition of PI3K using LY294002 (10 μM) eliminated the accumulation of the mitochondrial matrix protein HSP60 induced by the S protein, indicating that the inhibition of autophagy by the S protein is mainly mediated through the PI3K/Akt axis, rather than solely via the activation of mTOR (Fig. 3C).

We next explored whether the S protein directly interacts with LC3 to block its lipidation. Co-immunoprecipitation assays showed no detectable interaction between S and LC3 (Fig. S2A). To assess whether the S protein influences the fusion of autophagosomes with lysosomes, the binding of LC3 to p62 was detected by co-immunoprecipitation, and it was found that the S protein does not affect this fusion event (Fig. S2B). These results suggest that the S protein primarily disrupts autophagosome formation rather than autophagosome–lysosome fusion. Given the observed mitochondrial protein accumulation, we investigated whether the S protein associates with mitochondria. Mitochondrial fractionation from IBV-infected CEK cells confirmed the presence of the S protein within the mitochondrial fraction (Fig. 3D). Immunofluorescence microscopy confirmed partial colocalization of the S protein with TOMM20 and HSP60 (Fig. 3E and F), which could underlie its role in mitophagy suppression. However, compared with the empty vector control, S protein expression did not result in a significant alteration in mitochondrial membrane *ΔΨm* or reactive oxygen species (ROS) production (Fig. S2C and D). *ΔΨm* and ROS measurements revealed no evidence of mitochondrial dysfunction in S-expressing cells, indicating that mitophagy inhibition is not secondary to mitochondrial damage.

Mitophagy can be initiated via PINK1-dependent or receptor-mediated (PINK1-independent) pathways. Assessment of PINK1 levels within mitochondrial fractions showed that overexpression of the S protein did not affect PINK1 accumulation (Fig. 3G), suggesting a potential involvement of receptor-mediated mitophagy. Mitochondrial receptors, such as FUNDC1, NIX, BNIP3, FKBP8, and PHB2, contain both mitochondrial targeting sequences and LC3-interacting regions (LIRs) to facilitate mitophagy. We cloned and expressed each receptor in CEK cells to assess their potential interaction with the S protein. Co-IP assays revealed that the S protein specifically binds to FUNDC1 (Fig. 3H), a finding corroborated by both forward and reverse co-IP experiments (Fig. 3I; Fig. S2E). To elucidate the functional significance of FUNDC1 in S protein-mediated mitophagy inhibition, we silenced FUNDC1 in DF-1 cells using siRNAs. The knockdown efficiency was verified by qPCR, with siRNA152 achieving the most pronounced reduction in FUNDC1 transcripts (Fig. 3J). In FUNDC1-knockdown cells, the decrease in LC3-II levels mediated by the S protein disappeared (Fig. 3K), indicating that FUNDC1 plays a crucial role in inhibiting the autophagy mediated by the S protein.

### FUNDC1-mediated mitophagy promotes degradation of viral N protein and restricts IBV replication

**Fig 4:**
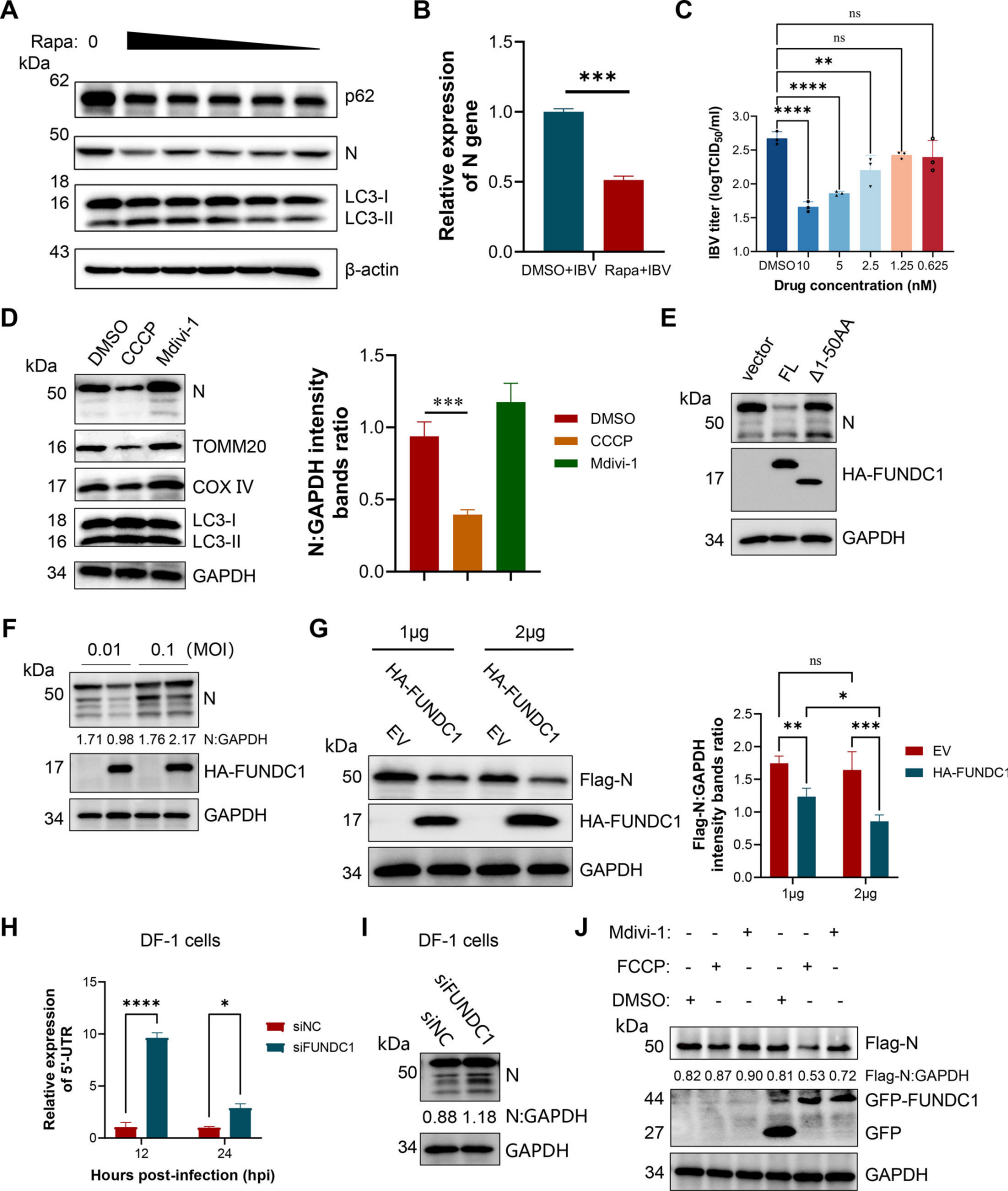
FUNDC1 inhibits IBV replication. (**A and C**) CEK cells were treated with rapamycin (10–0.625 nM) for 24 h and then infected with IBV at an MOI of 0.1 for 12 h. Cells and supernatants were collected, and N protein content and virus titer were measured, respectively. (**B**) Cell lysates were treated with rapamycin (10 μM) or DMSO for 24 h and then harvested for qPCR to test the relative expression of the N. (**D**) CEK cells were treated with CCCP (2 μM) or Mdivi-1 (5 μM) for 24 h and then infected with IBV at an MOI of 0.1 for 24 h. Cells and supernatants were collected, and N protein content and virus titer were measured, respectively; cell lysates were then harvested to detect N, TOMM20, and COX IV using western blotting. (**E**) CEK cells were transfected with HA-FUNDC1, HA-FUNDC1Δ1-50, or empty vector, followed by IBV infection. Cells were harvested at 24 hpi for western blotting. (**F**) CEK cells were transfected with HA-FUNDC1 or empty vector for 24 h and then infected with IBV (MOI = 0.01 or 0.1). Cells were harvested at 24 hpi for western blotting. (**G**) CEK cells seeded in 12-well plates were co-transfected with Flag-N (500 ng) with increasing doses of HA-FUNDC1 (300 or 500 ng) for 24 hpt; cell lysates were then harvested to detect Flag-N using western blotting. (**H**) DF-1 cells were infected with IBV for 12 or 24 hpi after transfection of siRNA or NC for 24 h, and 5′-UTR: β-actin was detected by qPCR. (**I**) DF-1 cells were treated the same as in panel H. Cells were harvested at 24 hpi for western blotting. (**J**) CEK cells seeded in 6-well plates were transfected with Flag-N (1 μg) together with empty vector or GFP-FUNDC1 (1 μg). At 12 hpt, cells were treated with DMSO or FCCP (2.5 μM) or Mdivi-1 (5 μM). After another 12 h, cells were harvested for western blot analysis with antibodies against Flag-tag, GFP-tag, and GAPDH. Data are mean ± SD from three independent experiments. Differences were considered significant at (∗) *P* < 0.05, (∗∗) 0.001 < *P* < 0.01, (∗∗∗) *P* < 0.001; ns, not significant.

We found that the induction of autophagy appears to suppress IBV replication in CEK cells. To further assess the functional importance of autophagy in viral replication, we developed an autophagy induction model using rapamycin. Treatment with 10 μM rapamycin for 24 h robustly induced autophagy without affecting cell viability (Fig. S3A and B). Under these conditions, rapamycin treatment significantly reduced IBV N protein expression (Fig. 4A), decreased N gene transcript levels in culture supernatants as measured by qRT-PCR (Fig. 4B), and lowered viral titers in a dose-dependent manner (Fig. 4C). Consistently, indirect immunofluorescence analysis revealed a pronounced reduction in IBV replication throughout the cell population (Fig. S3C).

We next examined whether selective mitophagy also influences IBV replication. Disruption of mitochondrial homeostasis with CCCP, a potent mitophagy inducer, markedly inhibited viral replication. In contrast, inhibition of mitochondrial fission with Mdivi-1 promoted viral growth (Fig. 4D). To determine whether the mitophagy receptor FUNDC1 is directly involved, we overexpressed HA-FUNDC1 in CEK cells. The full-length FUNDC1 strongly suppressed IBV replication, whereas an N-terminal truncation mutant lacking amino acids 1–50 failed to exhibit antiviral activity (Fig. 4E), indicating the critical role of this N-terminal region in FUNDC1 function. Notably, the inhibitory effect mediated by FUNDC1 was more pronounced at a lower infection dose (MOI = 0.01) compared to a higher dose (MOI = 0.1), suggesting that IBV may encode an antagonistic factor capable of counteracting FUNDC1 activity under high viral loads (Fig. 4F).

Considering that the IBV N protein has been proposed as a substrate for autophagic degradation (31), we next asked whether FUNDC1-mediated mitophagy influences N protein turnover. Overexpression of HA-FUNDC1 resulted in a dose-dependent reduction of N protein levels (Fig. 4G), whereas knockdown of FUNDC1 in DF-1 cells led to enhanced viral replication accompanied by increased N protein accumulation (Fig. 4H and I). Importantly, co-treatment with the mitochondrial stabilizer Mdivi-1 abolished the reduction of N protein levels mediated by FUNDC1 (Fig. 4J), indicating that N protein degradation requires intact mitochondrial dynamics and mitophagy. These results demonstrate that FUNDC1 restricts IBV replication by promoting mitophagy-dependent clearance of mitochondria associated with the viral N protein.

### The IBV S protein inhibits the interaction between FUNDC1 and LC3 by targeting the LIR motif of FUNDC1

Based on the observations in Fig. 4F, we hypothesized that the S protein might function as a regulatory switch that has evolved to counteract the antiviral activity of FUNDC1, potentially by promoting its degradation or interfering with its biological function. To evaluate this hypothesis, we first examined FUNDC1 expression following IBV infection. Western blot analysis revealed that infection with IBV did not alter FUNDC1 protein abundance in CEK cells (Fig. S4A). Similarly, FUNDC1 mRNA levels remained stable during 12–24 h of infection, irrespective of autophagy induction by rapamycin (Fig. S4B). These results indicate that the IBV exerts its effect by influencing the function of FUNDC1 rather than by regulating its expression.

**Fig 5:**
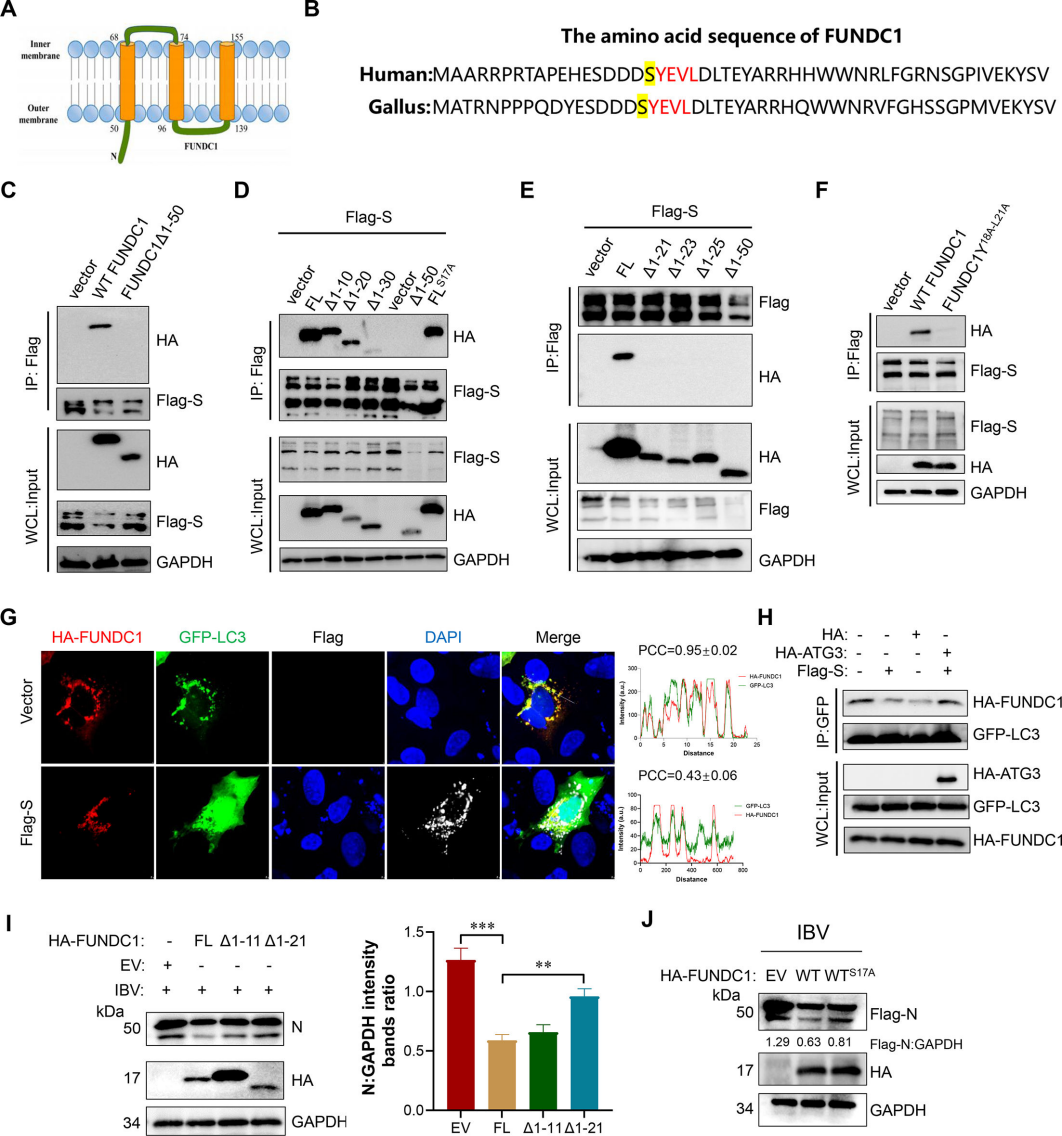
S protein impairs FUNDC1-LIR–mediated N degradation. (**A and B**) Schematic representation of the domain structure of FUNDC1 and alignment of the N-terminal 50 amino acids of avian and mammalian FUNDC1. (**C–F**) CEK cells were co-transfected with Flag-S protein and either HA-FUNDC1, its truncation, point mutations, or empty vector as indicated. Cell lysates were subjected to immunoprecipitation with anti-Flag antibody. (**G**) CEK cells were co-transfected with HA-FUNDC1, GFP-LC3, and Flag-S protein or empty vector. Colocalization of HA-FUNDC1 and GFP-LC3 was visualized by confocal microscopy at 24 h post-transfection. (**H**) CEK cells were co-transfected with HA-FUNDC1, GFP-LC3, Flag-S, and HA-ATG3 or empty vector. Immunoprecipitation was performed using anti-GFP antibody. (**I and J**) CEK cells were transfected with HA-FUNDC1 (wild-type, truncation, or point mutations) or empty vector for 24 h, followed by IBV infection for another 24 h. Viral N protein expression was examined by immunoblotting; GAPDH served as the loading control. Data are mean ± SD from three independent experiments. Differences were considered significant at (∗) *P* < 0.05, (∗∗) 0.001 < *P* < 0.01, (∗∗∗) *P* < 0.001.

Avian FUNDC1 shares high structural similarity with its mammalian counterpart, including an N-terminal mitochondrial outer membrane region encompassing the first 50 amino acids (Fig. 5A) and a conserved LC3-interacting region (LIR; YEVL motif) (Fig. 5B) (32). Previous research has demonstrated that phosphorylation of the serine residue immediately upstream of the LIR motif regulates its interaction with LC3 (14). However, we found that the S protein did not affect the phosphorylation at the SER18 position of FUNDC1 (Fig. S4C). Co-immunoprecipitation assays demonstrated that the S protein specifically interacts with the full-length FUNDC1 but not with N-terminally truncated forms (Fig. 5C). Detailed mapping localized the binding site to the region surrounding amino acid 20 of FUNDC1 (Fig. 5D and E), while no interaction was detected with the S17 residue alone. Mutation of the LIR motif (YEVL→AEVA) completely abolished S-FUNDC1 binding (Fig. 5F), confirming the LIR domain as the primary contact site for the S protein.

Given the critical role of the LIR motif in the FUNDC1–LC3 interaction and mitophagy, we next examined whether S protein binding interferes with this interaction. Using confocal microscopy and co-immunoprecipitation assays, we examined whether incorporation of the S protein affects the interaction between FUNDC1 and LC3. Immunofluorescence analysis showed that overexpression of the S protein reduced FUNDC1–LC3 colocalization, with the PCC decreasing from 0.95±0.02 to 0.43±0.06 (Fig. 5G). Co-IP analysis revealed that overexpression of the S protein markedly reduced FUNDC1–LC3 binding compared to vector controls, whereas this inhibition was substantially alleviated by co-expression of ATG3 (Fig. 5H). To improve the generalizability and recency of our results, we tested the S protein of the recently isolated IBV^63#^ strain. The IBV^63#^ S protein still interacted with FUNDC1 (Fig. S5A), consistent with the original strain, and similarly disrupted the FUNDC1–LC3 interaction (Fig. S5B). These findings indicate that the presence of the S protein restricts the interaction between FUNDC1 and LC3, thereby impairing the autophagic function of FUNDC1. To determine whether the antiviral activity of FUNDC1 is contingent upon its LIR motif, we generated a truncated mutant devoid of this domain. As shown in Fig. 5I, deletion of the LIR motif completely nullified FUNDC1’s capacity to inhibit IBV replication. Additionally, the S18A mutant of FUNDC1, which mimics dephosphorylation and reduces LC3 binding, displayed impaired ability to degrade the IBV N protein in comparison to wild-type FUNDC1 (Fig. 5J).

Taken together, these results demonstrate that FUNDC1 mediates the mitophagic degradation of the viral N protein through its LIR motif, and that the S protein antagonizes this antiviral pathway by competitively binding to the LIR domain and disrupting FUNDC1–LC3 coupling.

### Asparagine 240 of the S protein is critical for disrupting the interaction between FUNDC1 and LC3

**Fig 6:**
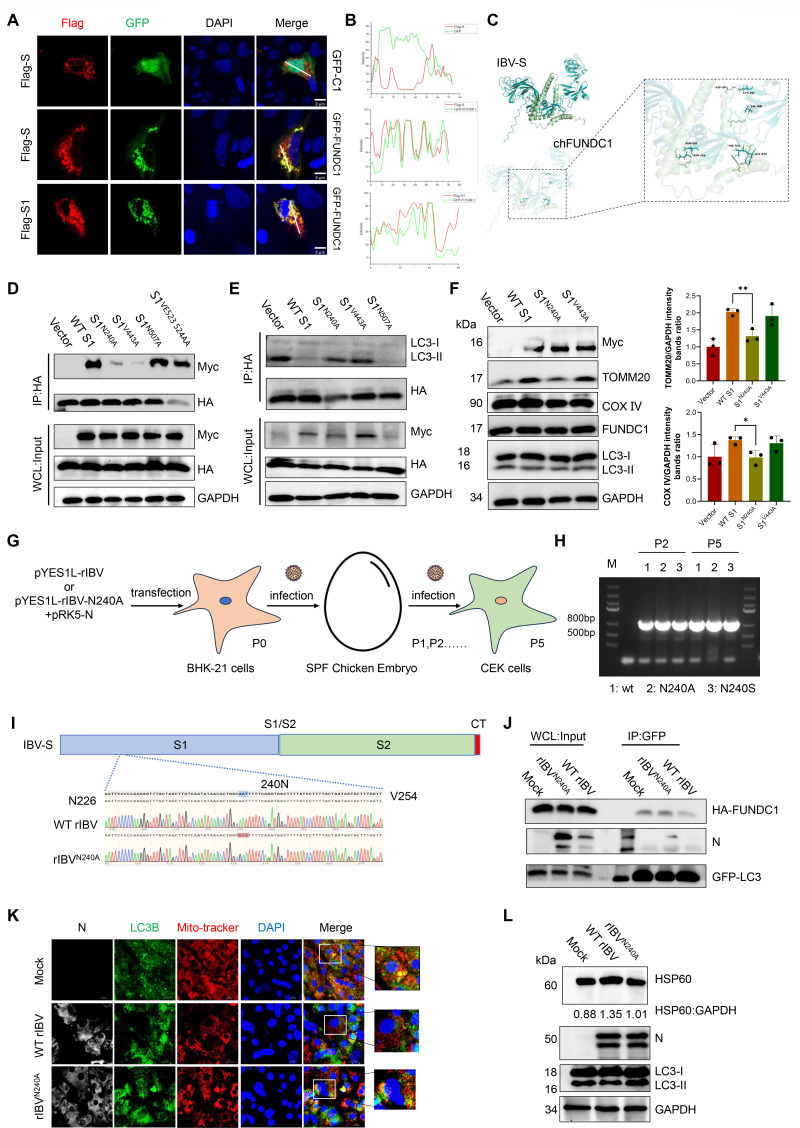
Asn240 modulates the FUNDC1–LC3 interaction. (**A and B**) CEK cells were co-transfected with Flag-S, Flag-S1, GFP-FUNDC1, or empty vector as indicated. Cells were subjected to confocal microscopy to assess colocalization between S/S1 and FUNDC1 24 hpt. (**C**) Molecular docking analysis predicting potential interaction sites between IBV S protein and FUNDC1. (**D**) CEK cells were co-transfected with HA-FUNDC1 and either wild-type S1 or the S1 point mutants. Cell lysates were subjected to immunoprecipitation using anti-HA antibody, and interactions between Myc-S1 and HA-FUNDC1 were detected by western blot. (**E**) CEK cells were treated as in panel D, and immunoprecipitation was performed using anti-HA antibody to detect the interaction between HA-FUNDC1 and LC3. (**F**) CEK cells were transfected with Myc-S1, Myc-S1^N240A^, Myc-S1^V443A^, or empty vector for 24 h. The levels of TOMM20, COX IV, and FUNDC1 were analyzed by western blot; GAPDH served as the control. (**G**) Schematic overview of the rescue strategy for generating mutant IBV strains via reverse genetics. (**H**) RT-PCR detection of the N gene from P5-passage allantoic fluid of rescued viruses. (**I**) Sequence alignment of the S gene amplified from the rescued viruses confirms successful mutation at the target site. (**J**) CEK cells were co-transfected with HA-FUNDC1 and GFP-LC3 for 12 h and then infected with WT rIBV or rIBV^N240A^ for an additional 24 h. Cell lysates were subjected to immunoprecipitation using anti-GFP antibody, and the interaction between GFP-LC3 and HA-FUNDC1 was analyzed by western blot. (**K**) CEK cells were infected with rIBV, rIBV^N240A^, or mock for 24 h. Mitochondria were labeled with Mito-Tracker, and colocalization between LC3B and mitochondria was assessed by confocal microscopy. (**L**) CEK cells were treated as in panel K, and HSP60 levels were analyzed by western blot; the HSP60:GAPDH ratio was quantified. Data are mean ± SD from three independent experiments. Differences were considered significant at (∗) *P* < 0.05, (∗∗) 0.001 < *P* < 0.01, (∗∗∗) *P* < 0.001.

Previous studies have shown that the receptor-binding domain (RBD) and S1 subunit of coronaviruses can suppress mitophagy (21). To delineate the key residues mediating the interaction between the IBV S protein and FUNDC1, we first performed confocal microscopy and found that the S1 subunit predominantly colocalizes with FUNDC1 (Fig. 6A and B). Molecular docking analysis, based on homology modeling of the IBV S protein, predicted five potential interaction sites with FUNDC1 (Fig. 6C). Site-directed mutagenesis of these predicted residues followed by co-immunoprecipitation revealed that asparagine residues at positions 240 (N240) and 443 (N443) are essential for the interaction with FUNDC1 (Fig. 6D). Notably, mutation at either site enhanced FUNDC1’s binding to LC3 (Fig. 6E), but only the N240A mutation was sufficient to restore mitochondrial content to levels comparable to the empty vector in CEK cells (Fig. 6F), indicating that the S protein residue N240 plays a critical role in both structural and functional interactions with FUNDC1. We next assessed the evolutionary conservation of N240 across various IBV strains. Sequence analysis of over 800 IBV S protein sequences from the NCBI database revealed that N240 resides within a highly conserved region (Fig. S5C). To assess the functional importance of residue N240 during viral infection, a recombinant IBV carrying the N240A mutation was generated using a reverse genetics system (Fig. 6G). The rescued virus was successfully propagated in embryonated chicken eggs, and viral RNA was readily detected by PCR after serial passages (Fig. 6H). Sequencing of the S gene from passage 5 confirmed the presence of the intended N240A substitution without additional mutations (Fig. 6I).

To evaluate the functional consequences of the mutation, the CEK cells were infected with either the wild-type or N240A-mutant IBV. Co-immunoprecipitation assays showed that the N240A mutant virus restored the interaction between FUNDC1 and LC3, which was disrupted by the wild-type IBV (Fig. 6J). Confocal imaging further revealed that LC3 colocalization with mitochondria was markedly increased in cells infected with the N240A mutant compared to those infected with the wild-type virus (Fig. 6K). Moreover, the levels of the mitochondrial marker protein HSP60 were partially restored in cells infected with the N240A mutant, suggesting that the inhibition of mitophagy was relieved (Fig. 6L). Together, these results indicate that the IBV S protein impairs mitophagy by binding to the LIR domain of FUNDC1 via residue N240.

### The N240A mutation attenuates IBV pathogenicity by restoring mitophagy *in vivo*

**Fig 7:**
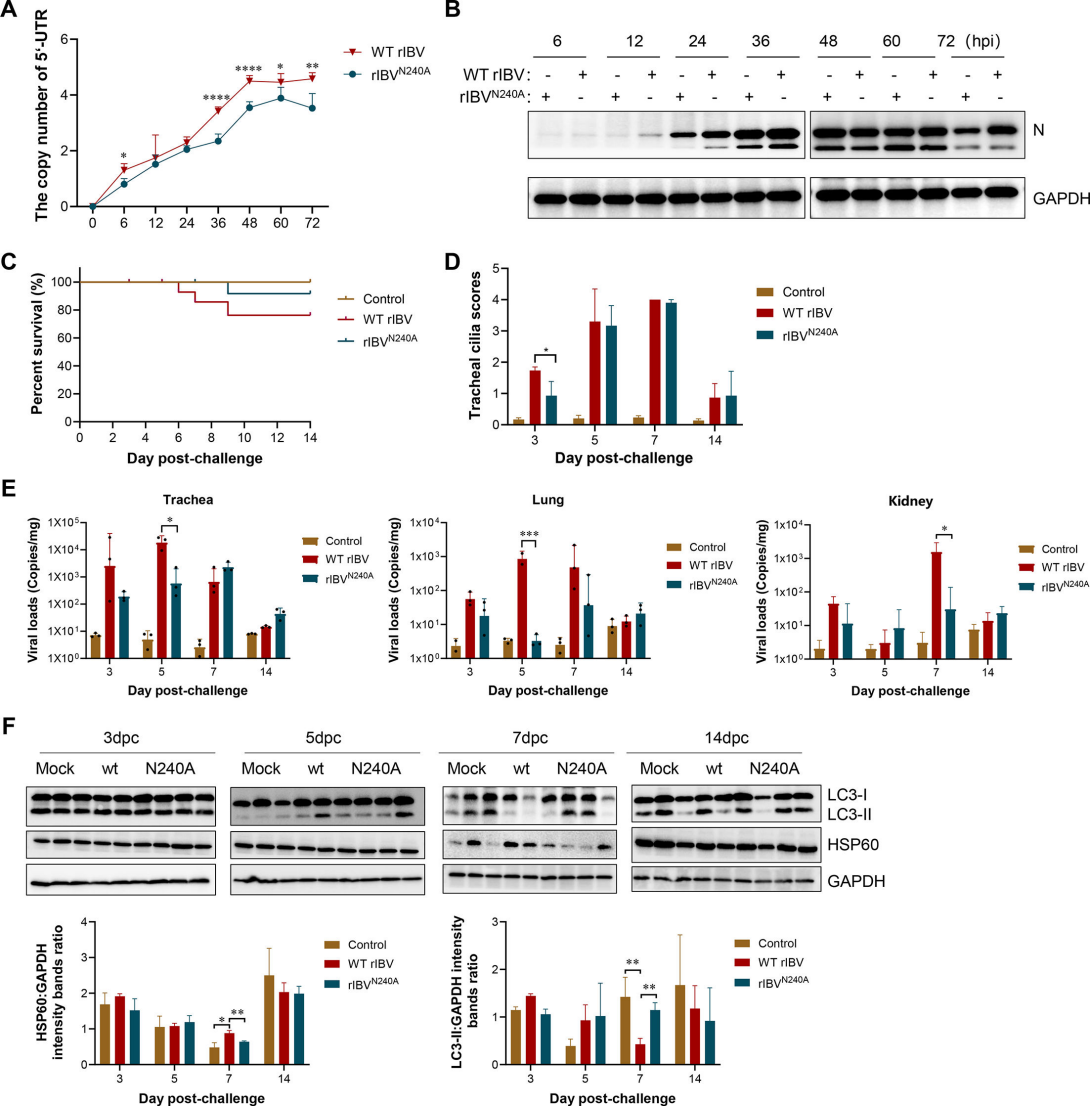
The N240A mutation attenuates IBV pathogenicity. (**A**) Replication kinetics of rIBV^N240A^ compared with parental strain in CEK cells. The cell supernatants were collected at different time points after infection to quantify viral RNA copy numbers via qRT-PCR. (**B**) The expression level of the virus N protein is detected via western blotting. (**C**) Survival curves of 1-day-old SPF chickens infected with rIBV^N240A^ compared with WT rIBV. (**D**) Ciliostasis scores of 1-day-old SPF chickens infected with rIBV^N240A^ compared with WT rIBV. Ciliary activity was scored based on the following criteria: 0 = 100% ciliary activity; 1 = 75–100% ciliary activity; 2 = 50–75% ciliary activity; 3 = 25–50% ciliary activity; and 4 = 0–25% ciliary activity. (**E**) Tissue viral loads of 1-day-old SPF chickens infected with rIBV^N240A^ compared with WT rIBV. Viral loads in the trachea, lungs, and kidneys of chickens at 3, 5, 7, and 14 days post-infection with IBV were determined by quantitative real-time PCR. (**F**) Autophagy levels in kidney tissues of 1-day-old SPF chickens infected with rIBV^N240A^ compared with WT rIBV. Data are mean ± SD from three independent experiments. Differences were considered significant at (∗) *P* < 0.05, (∗∗) 0.001 < *P* < 0.01, (∗∗∗) *P* < 0.001.

To verify the impact of the interaction between the S and FUNDC1 on the pathogenicity of IBV, we assessed the replication kinetics of both wild-type and N240A-mutant viruses in CEK cells over a period of 0 to 72 hpi. The N240A mutant exhibited significantly reduced replication compared to the wild-type virus (Fig. 7A and B), suggesting that the interaction between the S protein and FUNDC1 facilitates viral replication. S protein mediates receptor binding and viral entry. Binding and entry assays showed no significant differences between the WT rIBV and rIBV^N240A^ mutant viruses, indicating that the N240A mutation does not impair viral attachment or entry (Fig. S6A and B). In contrast, single-cycle replication kinetics revealed markedly reduced replication of the mutant virus, suggesting that the attenuated phenotype is primarily attributable to a defect in viral replication rather than to impaired entry (Fig. S6C).

We next evaluated the *in vivo* relevance of this interaction in an SPF chicken infection model. One-day-old SPF chicks were inoculated with either WT rIBV or rIBV^N240A^ and monitored over a 14-day period. Post-mortem analyses were performed on days 3, 5, 7, and 14, with collection of trachea, lung, and kidney samples—key target organs of IBV. Chicks infected with WT rIBV exhibited more severe clinical signs and a mortality rate of 23.9%, while those infected with the rIBV^N240A^ mutant showed milder symptoms and a reduced mortality rate of 8.4% (Fig. 7C). At 3 dpi, tracheal ciliary activity scores were significantly lower in the WT group than in the mutant group (Fig. 7D). Gross pathology showed that birds infected with WT rIBV developed severe lesions, characterized by multiple hemorrhagic spots in the tracheal mucosa and larynx, pulmonary necrotic foci, and pale, mottled kidneys. In contrast, only minor pinpoint hemorrhages were observed in the larynx of birds infected with the rIBV^N240A^ mutant (Fig. S7A). Histopathological examination further revealed extensive mucosal atrophy and epithelial loss in the trachea, pulmonary congestion and hemorrhage, and widespread tubular necrosis with inflammatory infiltration in the kidneys of the WT group. In contrast, the N240A mutant caused only mild epithelial desquamation in the trachea and moderate renal swelling (Fig. S7B).

RT-qPCR analysis of viral RNA levels in the trachea, lung, and kidney revealed that the rIBV^N240A^ group harbored lower viral loads than the WT rIBV group across all tissues examined (Fig. 7E). To explore the potential impact of the mutation on mitophagy *in vivo*, we assessed autophagy markers in kidney tissue. At 7 dpi, LC3-II levels were significantly elevated, and the mitochondrial marker HSP60 was partially restored in the rIBV^N240A^ group relative to the WT rIBV group (Fig. 7F), indicating that the disruption of the S-FUNDC1 interaction attenuates the virus’s capacity to suppress mitophagy in target organs. Taken together, these findings demonstrate that disruption of the S protein–FUNDC1 interaction attenuates IBV virulence *in vivo*.

## DISCUSSION

**Fig 8:**
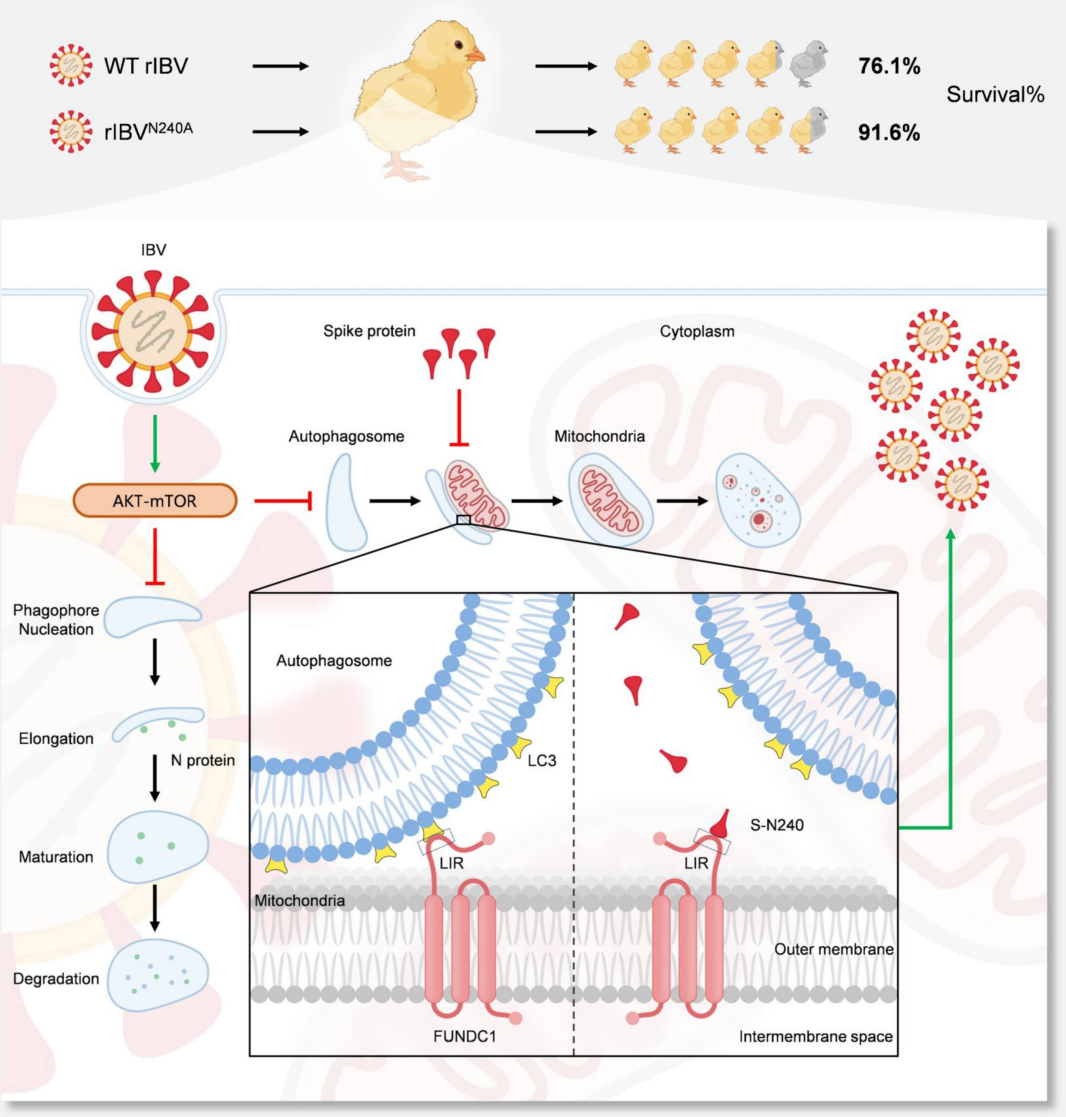
A proposed model illustrating how spike protein of IBV evades FUNDC1-mediated antiviral responses.

Autophagy is a critical cellular defense mechanism triggered by various stimuli, including nutrient deprivation, protein misfolding, organelle damage, and pathogen invasion (33–35). Our study reveals a novel strategy by which coronaviruses subvert host mitophagy to evade antiviral responses. Specifically, we demonstrate that FUNDC1-mediated mitophagy promotes degradation of viral N protein and restricts IBV replication. In response, the viral S protein counteracts this restriction by directly interacting with the mitochondrial outer membrane protein FUNDC1. Mechanistically, the S protein competes with LC3 for binding to the LIR motif of FUNDC1, thereby impairing FUNDC1–LC3 interaction, suppressing mitophagy, and weakening FUNDC1-mediated antiviral effects. We identified asparagine at position 240 (N240) of the S protein as a key residue responsible for this interaction. Using reverse genetics and yeast-based recombination, we rescued a mutant strain harboring the N240A substitution. Notably, this mutant virus exhibited enhanced mitophagy and reduced pathogenicity in SPF chickens compared to the wild-type strain (Fig. 8).

Previous studies using the Beaudette strain in Vero cells suggested that IBV non-structural protein 6 (NSP6) induces autophagosome formation via ER-derived omegasome intermediates (36). However, later findings indicated that in avian cells, NSP6 may instead restrict autophagosome expansion, facilitating viral replication by hindering auto-lysosomal degradation of viral components (24). These contradictory results underscore the limitations of extrapolating findings from mammalian cell lines to the natural avian host of IBV. The AKT–mTOR pathway is a classic autophagy signaling pathway, and flaviviruses also regulate cellular autophagy through this pathway (37, 38). Our data showing AKT–mTOR activation during IBV infection provide a plausible explanation for why NSP6 fails to induce autophagy during viral infection. Although rapamycin restored LC3-II accumulation during IBV infection, it failed to rescue mitophagy or clear accumulated mitochondria in S protein-expressing cells (Fig. S8). This indicates that the S protein inhibits FUNDC1-dependent mitophagy independently of, and cannot be overcome by, mTOR inhibition. We propose that virus-induced AKT–mTOR activation broadly suppresses autophagy to preserve membrane resources for double-membrane vesicle formation. This is also currently under investigation in our laboratory.

Consistent with earlier observations that IBV does not induce significant autophagosome accumulation in avian cells (23), we show that infection with field strains of IBV in CEK cells markedly inhibits autophagosome formation and promotes mitochondrial accumulation. Similar autophagy suppression has been observed with α-coronaviruses such as porcine epidemic diarrhea virus (PEDV) and transmissible gastroenteritis virus (TGEV) in porcine intestinal epithelial cells, suggesting a conserved strategy among α- and γ-coronaviruses to inhibit autophagy in their natural target cells, possibly due to their dependence on host membrane structures for replication (37, 39). The ORF7a protein of SARS-CoV-2, although capable of inducing autophagy, blocks autophagic flux and thereby promotes viral replication (40). The diverse mechanisms by which coronaviruses manipulate cellular autophagy suggest that autophagy may not be essential for coronavirus replication. We speculate that these differences are more likely associated with variations in the pathogenicity of different coronavirus strains.

Coronaviruses have been reported to induce both mitochondrial dysfunction and mitophagy impairment (19, 41, 42). Proteomic analyses of host factors interacting with the IBV S1 protein have identified 127 candidates, predominantly enriched in pathways associated with cell adhesion, cell death, and autophagy (30). MAO-B, a flavin-containing enzyme located on the mitochondrial outer membrane that catalyzes the oxidative deamination of monoamine neurotransmitters, has attracted particular attention (43). The SARS-CoV-2 spike glycoprotein has been shown to interact with MAO-B, resulting in elevated enzymatic activity in SH-SY5Y neuroblastoma cells, perturbations in neurotransmitter metabolism, and potentially contributing to coronavirus-associated neurodegenerative pathology (22). These findings support the hypothesis that the disruption of mitochondrial homeostasis by coronavirus S proteins may represent an evolutionarily conserved strategy (44). In addition to S proteins, other coronavirus-encoded factors, such as the accessory protein ORF10, have been implicated in remodeling mitochondrial metabolism (45). Our previous IBV protein screening further suggested that several non-structural proteins, including NSP3 and NSP8, may also exert autophagy-inhibitory effects, possibly linked to the formation of double-membrane vesicles during viral replication. Future work will focus on elucidating the molecular mechanisms by which multiple IBV proteins coordinately manipulate host autophagy, thereby refining our understanding of the diverse strategies coronaviruses employ to evade cellular defense mechanisms.

The intricate interactions between viruses and their hosts have long been a focus of virology. Most studies have focused on how viruses counteract innate immune response, often with autophagy receptors playing an indirect role. For instance, SARS-CoV-2 ORF10 has been shown to interact with the mitophagy receptor NIX to promote MAVS suppression via mitophagy (46). Similarly, the pseudorabies virus envelope protein UL21 triggers cGAS degradation, thereby attenuating type I interferon signaling (47). In contrast, our study showed that avian FUNDC1 directly restricts IBV by mediating the autophagic degradation of the N protein. Although complete genetic ablation of FUNDC1 in CEK cells is not feasible, knockdown experiments clearly underscore its antiviral function. Our findings demonstrate that the S protein directly interacts with FUNDC1, thereby blocking LC3 recruitment—a key step in mitophagosome initiation—and consequently leading to the accumulation of dysfunctional mitochondria. Similar antagonistic strategies are observed in other viral infections. During Sindbis virus infection, SQSTM1/p62 delivers the capsid protein to autophagosomes for degradation (48), while NBR1 targets the PDCoV envelope protein for selective autophagy. In turn, the PDCoV NSP5 protease cleaves NBR1, thereby disabling its antiviral activity (20). Similar to SARS-CoV-2, which modulates mitochondrial dynamics via its ORF10 and NSP5 proteins, IBV appears to employ its S protein to interfere with mitophagy—a finding suggesting that coronaviruses have undergone convergent evolution in targeting mitochondrial function.

It is well-established that IBV infection can cause severe acute kidney injury (AKI) (49). Previous studies have reported that viruses can induce inflammation by suppressing mitophagy and enhancing mitochondrial reactive oxygen species (mtROS) production. For instance, the NS5 protein of the Zika virus (ZIKV) antagonizes mitophagy by binding to the host protein Ajuba, thereby preventing its translocation to depolarized mitochondria. This interaction amplifies the proinflammatory chemokine production and enhances tissue tropism (50). Similarly, the hepatitis C virus (HCV) core protein suppresses mitophagy by inhibiting the translocation of Parkin to mitochondria, which may exacerbate and sustain HCV-induced mitochondrial damage, ultimately leading to severe tissue injury (51). Collectively, these findings suggest that a variety of viruses can target mitochondria and disrupt mitochondrial network homeostasis, thereby altering host cell metabolism and physiology to facilitate immune evasion, enhance tissue tropism, and maintain persistent infection. The mitophagy receptor FUNDC1 plays a critical role in preserving mitochondrial function and in the pathogenesis of kidney diseases (52). Therefore, investigating the role of inhibited mitophagy in IBV-induced renal injury will be a key direction for our future research.

However, our study has certain limitations. To better recapitulate natural infection, we employed CEK cells, which made gene knockdown and knockout approaches technically challenging. In the future, this limitation could be addressed by developing immortalized cell lines or by rescuing IBV strains adapted to commonly used cell lines. In addition, the molecular mechanisms underlying the differential effects of IBV on autophagy in avian versus mammalian cells remain to be elucidated. This question can be investigated using advanced viral tracking technologies or a broader repertoire of avian organelle-specific markers. Moreover, since the S protein predominantly localizes within the endoplasmic reticulum, how it establishes contact with mitochondria—and particularly the role of the key mitochondria-associated membrane (MAM) protein FUNDC1 in this process—remains an important direction for our future research.

Collectively, our findings identify the S-FUNDC1 axis as a critical viral-host interface, offering potential targets for antiviral intervention aimed at restoring mitophagy and limiting coronavirus replication. These findings not only advance our understanding of γ-coronavirus–host interactions but also suggest that targeting the S-FUNDC1 interactions might offer a potential therapeutic strategy for coronavirus-induced acute kidney injury.

## MATERIALS AND METHODS

### Cells and viruses

CEK cells were prepared from 18-day-old SPF chicken embryos as previously described (53). SPF embryonated eggs were purchased from Beijing Boehringer Ingelheim Vital Biotechnology Co., Ltd. (Beijing, China). DF-1 cells (ATCC, CRL-3586) and HEK-293T cells (ATCC, CRL-3216) were grown at 37°C with 5% CO₂ in Dulbecco’s Modified Eagle’s Medium (DMEM; Gibco 12800082) supplemented with 10% fetal bovine serum (FBS; Gibco 16000044). The IBV strains YN (GenBank: JF893452.2) and SD (GenBank: KY421673) were used in this study. After infection, IBV-infected CEK cells were cultured for the required incubation period followed by two freeze/thaw cycles. The supernatant was collected, and IBV titer was determined by tissue culture infectious dose 50 (TCID_50_) of CEK cells.

### Generation of mutant viruses

Virus rescue experiments were conducted as previously described (54). In brief, confluent monolayers of BHK-21 cells (10^6^ cells per well in six-well plates) were transfected with 2.5 μg per well of IBV YAC-BAC shuttle plasmid using Lipofectamine 2000 (Thermo Fisher Scientific). Following a transfection period of 6 h, the transfection medium was replaced with post-infection medium (DMEM supplemented with 2% FBS and 1% PSG). After 48 h post-transfection, the cells were cryopreserved at −80°C and designated as passage 0 (P0). Cells underwent three freeze/thaw cycles before the lysates were inoculated into the allantoic cavities of 9- to 11-day-old SPF chicken embryos. Allantoic fluid was harvested 48 h post-inoculation for further passages.

### Virus growth kinetics

Virus growth kinetics were analyzed in both CEK cells and embryonated chicken eggs (ECEs). The CEK cells were infected with 10^7^ viral genome copies. After 1 h of adsorption, surface virions were removed by washing the cells three times with PBS. To evaluate replication curves in ECEs, 10-day-old embryos were inoculated with 10^5^ viral genome copies, and cell supernatants or allantoic fluids were collected at specified intervals (6, 12, 24, 36, 48, 60, and 72 hpi). Viral RNA was extracted using the Hipure RNA Mini Kit (Magen, Beijing, China), reverse-transcribed with the Star Script III All-in-One RT Mix with gDNA Remover Kit (Genstar, Beijing, China), and quantitative real-time PCR was performed using the Light Cycler 96 with 2× M5 Hiper SYBR Premix Es Tag (Mei5bio, Beijing, China). The primers were designed targeting the conserved 5′ UTR of IBV (qpF: 5′-GTTGGGCTACGTTCTCGC-3′, qpR:5′-AAGCCATGTTGTCACTG TCTAT-3′). Copy numbers (*x*) were calculated from Ct values based on a standard curve (Ct = −3.41 × log10^x^ + 39.1).

### Animal experiment design

SPF chickens were randomly divided into challenge and control groups. Birds in the challenge group were inoculated via the oculonasal route with 200 μL of allantoic fluid containing 10^5^ EID_50_ of IBV. Birds in the control group were administered 200 μL of saline solution via the oculonasal route. At 3, 5, 7, and 14 days post-challenge (dpc), three birds from each group were euthanized and necropsied. Tissue samples from the trachea, kidney, and lung were collected for virus detection by a real-time quantitative reverse transcription-polymerase chain reaction (RT-qPCR) assay. All surviving chickens were humanely euthanized at the end of the experiment.

### Ciliary activity scoring of the trachea

The trachea of each chicken was excised and divided into upper, middle, and lower sections. From each section, 3–4 complete tracheal rings, approximately 1 mm in width, were collected, totaling 10 rings per bird. The tracheal rings were immediately placed into a 96-well culture plate containing 100 μL of DMEM per well. Ciliary movement was examined under a light microscope, and the extent of ciliary activity was scored based on the following criteria: 0 =100% ciliary activity; 1 = 75–100% ciliary activity; 2 = 50–75% ciliary activity; 3 = 25–50% ciliary activity; and 4 = 0–25% ciliary activity. The ciliary activity score for each individual bird was then calculated as the mean value of all 10 tracheal rings examined.

### RNA sequencing and enrichment analysis of the differentially expressed genes annotation

CEK cells were infected with IBV for 24 h, and then, RNA-seq transcriptome analysis was performed (*n* = 3). Total RNA was extracted using Trizol reagent kit (Invitrogen, Carlsbad, CA, USA) according to the manufacturer’s protocol. The resulting cDNA library was sequenced using Illumina Novaseq6000 by Gene Denovo Biotechnology Co. (Guangzhou, China). Adapter sequences and low-quality reads were filtered and trimmed using fastp (version 0.18.0). The short-read alignment tool Bowtie2 (version 2.2.8) was used to map reads to ribosome RNA (rRNA) database. The rRNA-mapped reads will then be removed. The remaining clean reads were further used for assembly and gene abundance calculation. The resulting non-ribosomal reads were mapped using Bowtie2 (v2.02, –sensitive local), with the best mapping locations reported for each read. An index of the reference genome was built, and paired-end clean reads were mapped to the reference genome using HISAT2 2.1.0, with all other parameters set as a default. RNAs’ differential expression analysis was performed by DESeq software between two different groups. Genes/transcripts with the parameter of false discovery rate (FDR) below 0.05 and absolute fold change≥2 were considered differentially expressed genes/transcripts. Functional enrichment of Gene Ontology (GO) terms was performed using Omicsmart tools (https://www.omicsmart.com).

### Antibodies and chemical reagents

Primary antibodies used for detecting viral and host proteins included anti-IBV N monoclonal antibody (Hytest, Finland); anti-HA (3724S), anti-β-actin (4970T), anti-AKT (4691T), anti-p-AKT (4060S), anti-mTOR (2983T), and anti-p-mTOR (5536T) (all from Cell Signaling Technology); anti-GAPDH (AC035) and anti-HSP60 (A0564) (both from ABclonal); anti-LC3 (L8918) and anti-p62 (P0067) (both from Sigma, San Francisco, CA, USA); and anti-TOMM20 (11802-1-AP) and anti-FUNDC1 (28519-1-AP) (both from Proteintech). Protein signal intensities were normalized and quantified using ImageJ software. All drugs used in this study—rapamycin (HY-10219), bafilomycin A1 (HY-100558), FCCP (HY-100410R), CCCP (HY-100941), and LY294002 (HY-10108)—were obtained from Med Chem Express (MCE, Princeton, New Jersey, USA).

### Plasmids

The full-length gallus FUNDC1 cDNA (NCBI Reference Sequence: NM_001276363.2) was amplified by reverse transcription RT-PCR from total RNA extracted from CEK cells and subsequently cloned into a modified pCAGGS vector with a HA tag at the C terminus and pCAGGS-GFP vectors. The truncated FUNDC1 mutants, including FUNDC1-N and Δ1-50 FUNDC1, were cloned into the pCAGGS vector with a HA tag at the C terminus. The IBV genes encoding viral structural proteins (S, E, M, N, etc., GenBank: KY421673) were separately cloned into the pRK5 vector with a Flag tag at the N-terminus using standard molecular biology techniques. The S1 mutants were generated by Mut Express II Fast Mutagenesis Kit V2 (Vazyme, NanJing, CHINA). The FUNDC1 mutants (FUNDC1^S17A^, FUNDC1^Y18AEVL21A^) were generated by site-directed mutagenesis using overlap extension PCR and subsequently cloned into the pCAGGS-HA vectors. The tandem fluorescent monomeric green fluorescent protein GFP-LC3 and pCAGGS were stored in our laboratory. All plasmid constructs were verified by DNA sequencing. PlusTrans Transfection Reagent (Nulen, Shanghai, China) was used for transient transfection of plasmids into CEK, HEK-293T, or DF-1 cells. The primers used are listed in Table S1.

### siRNA oligonucleotide transfection

For the RNA interference knockdown experiments, siRNA (sense strand-only shown) against gallus FUNDC1 (sense, 5′-GGCCGGAAUUCAGGACCAATT-3′; antisense, 5′-UUGGUCCUGAAUUCCGGCCTT-3′) was synthesized from Suzhou GenePharma. DF-1 cells were transfected with 60 nM (final concentration) siRNA using Lipofectamine RNAiMAX (Thermo Fisher Scientific, 13778030) following the manufacturer’s protocols. At 24 h post-transfection with siRNA, cells were further transfected with indicated plasmids with PlusTrans Transfection Reagent for another 24 h. Negative control siRNA (sense, 5′-UUCUCCGAACGUGUCACGUTT -3′; antisense, 5′-ACGUGACACGUUCGGAGAATT-3′) from Suzhou GenePharma was used as the control.

### Quantitative reverse transcription-PCR (RT-qPCR)

Total RNAs were extracted from cells using the GeneJET RNA Purification Kit (Thermo Scientific, Waltham, MA, USA) and reverse-transcribed using the ReverTra Ace qPCR RT Kit (Toyobo, Osaka, Japan) according to the manufacturers’ instructions. Relative quantitative PCR (qPCR) was performed to quantify IBV N gene expression, with GAPDH as the internal reference. Reactions (20 μL total volume) contained 1× SYBR Green Real-Time PCR Master Mix (Toyobo, Osaka, Japan), 0.4 μM each primer, and 2 μL of diluted cDNA (equivalent to ~20 ng input RNA). All qPCR assays were run on a LightCycler 96 instrument (Roche, Basel, Switzerland) using the following cycling conditions: initial denaturation at 95°C for 60 s, followed by 40 cycles of 95°C for 15 s (denaturation) and 60°C for 30 s (annealing/extension, with fluorescence acquisition). A melting curve analysis (65–95°C, 0.1°C/s ramp rate) was performed after amplification to confirm specificity of the PCR products.

### SDS-PAGE and western blot

Cells were lysed with cell lysis buffer for western blotting and IP (Beyotime, P0013) containing protease inhibitor (Roche, 04693132001). The supernatants were collected, and equal amounts of proteins were separated by SDS-PAGE and then were transferred to a nitrocellulose membrane (Bio-Rad, 162-0177). After blocking with 2% bovine serum albumin (BSA; Sigma, A1933) in Tris-buffered saline with Tween (TBST; Sigma, SRE0031), the membrane was incubated with the indicated primary antibodies, followed by HRP-conjugated secondary antibodies, and signals were detected by using a Western Bright ECL Detection Kit (Beyotime, P0018M) in an ECL detection system (Amersham Biosciences, Piscataway, NJ, USA). All bands of western blots were detected within the linear range.

### Co-immunoprecipitation (co-IP)

After transfection with the indicated plasmids, cells were washed three times with ice-cold PBS (HyClone, SH30256.01) and lysed in 500 μL ice-cold RIPA lysis buffer (Beyotime, P0013B) supplemented with protease inhibitors for 30 min. The cell lysates were clarified by centrifugation at 13,000 × *g* for 10 min. The supernatant was then incubated with specific antibodies for 8 h at 4°C with agitation, followed by an additional 2-hour incubation with Protein A/G Plus-Agarose (Santa Cruz Biotechnology, sc-2003). The beads were collected by centrifugation at 2,500 × *g* for 5 min at 4°C, washed four times with cold PBST, and the proteins were eluted by boiling in SDS loading buffer for 10 min. Finally, the proteins were analyzed using standard immunoblotting techniques.

### Immunofluorescence analyses

CEK cells were seeded on coverslips and co-transfected with pEGFP-LC3 and empty vectors. At 24 hpt, the cells were fixed with 4% formaldehyde and permeabilized with 0.1% (vol/vol) Triton X-100 in PBS and subsequent blocking with PBS buffer containing 1% fetal calf serum. The transfected CEK cells were incubated overnight at 4°C with the primary antibody, followed by incubation at room temperature for 1 h with the corresponding secondary antibody conjugated to Alexa-488/555 (Life Technologies, Gaithersburg, MD, USA). Fluorescence signals were captured using an A1 confocal microscope (Nikon, Tokyo, Japan). PCC is commonly employed to assess the spatial colocalization of two fluorescently labeled molecules or structures within a cell. The PCC values range from −1 to +1, where +1 indicates a perfect positive correlation (complete colocalization), 0 indicates no correlation (random distribution), and −1 indicates a perfect negative correlation.

### Transmission electron microscopy (TEM) assay

CEK cells grown to 70%–80% confluence in 6-well plates were infected with PBS or IBV, respectively, and treated by rapamycin. The different treatments in each group were collected and centrifuged for 5 min at 800–1,000 r/min. The supernatant was discarded, the cell sediment at the bottom of the centrifuge tube was collected, and the buffer was added. After being centrifuged for 5 min at 5,000 r/min, the dense adherent cell pellet was collected. The cell pellets were fixed with 2–4% glutaraldehyde (or a paraformaldehyde-glutaraldehyde mixture), and the fixed samples were sent to Wuhan Baiqiandu Technology Co., Ltd. (Wuhan, China) for TEM processing and observation. Images were acquired on a JSM-7500F transmission electron microscope (JEOL, Tokyo, Japan).

### TCID_50_ assay

CEK cells grown in a 96-well plate were infected with 0.1 mL/ well of 10-fold serially diluted supernatants in quintuplicate. After incubating for 120 min at 37°C, unattached virus was removed, and DMEM supplemented with 1% FBS was added to the cells. Four days post-infection, TCID_50_ was determined using the Reed-Muench method. All data are presented as means of three independent experiments.

### Statistical analysis

The results are presented as means ± SD. Student’s *t*-test was used to compare the data between the treated and control groups. Statistical significance is indicated by asterisks (*P < 0.05; **P < 0.01; ***P < 0.001; ns, not significant). All statistical analyses and calculations were performed using GraphPad Prism 10.3.1.

## Data Availability

All data supporting the findings of this study are provided within the article and supplemental material.

## SUPPLEMENTAL MATERIAL

Supplemental material jvi.01800-25-s0001.pdf Table S1; Fig. S1 to S8.

ASM does not own the copyrights to Supplemental Material that may be linked to, or accessed through, an article. The authors have granted ASM a non-exclusive, world-wide license to publish the Supplemental Material files. Please contact the corresponding author directly for reuse.
