# Supplementary material for: Coronavirus infectious bronchitis virus spike protein inhibits FUNDC1-mediated mitophagy to prevent nucleocapsid protein degradation
Source: J Virol. 2026 Apr 20;100(5):e01800-25. doi: 10.1128/jvi.01800-25 (PMC13185597; doi:10.1128/jvi.01800-25)
Supplement: Supplemental material — Table S1; Fig. S1 to S8. [file jvi.01800-25-s0001.pdf]

Supplementary Material

TABLE S1 Sequences of primers used for qPCR detection.

Name	Sense (5'-3')	Antisense (5'-3')
IBV-N	AAAAGGGTTCCCGCATTACAAA	ATTGCCGTAACACGCCCATC
ATG3	AAGGTGCAAGCAGATGGAGT	TCTTCACAGGATGCTGATCG
ATG5	TTCGAGATGTGTGGTTTGGA	CCATTTCAGTGGCGTACCTT
ATG7	CAGGCCATCCAGTGAATTTT	TGCCAGTTTCTTGCTGTTTG
ATG13	TCCTGATTCCCCAGAAACTG	CAAATCCTCTGCCATGGACT
GAPDH	ATCACAGCCACACAGAAGACG	TGACTTTCCCCACAGCCTTA
HA-FUNDC1	CTGTGCTGGATATCTGCAGAATTC ATGGCGGCGCGGAGGCCCCGCACC	ATGAGTTTTTGTTCTAGAAAGCTT TTACGACGCAAGGCCCAACAAAAAG
qFUNDC1	ACGGAATATGCAAGGCGTCA	GCTGCCTTATTTGCACGCTT
GFP-LC3	CTCGGCATGGACGAGCTGTACAAG ATGCCCTCGGAGAAGAGCTT	CGAGCGGCCGCCACTGTGCTGGAT CTAGACGGAAGATTGCACTC
GFP-FUNDC1	CTCGGCATGGACGAGCTGTACAAG ATGGCGGCGCGGAGGCCCCG	CGAGCGGCCGCCACTGTGCTGGA TAGTTACGACGCAAGGCCCAAC
Myc-S1	ATCCACTAGTCCAGTGTGGTGGAATTC ATGTTGGGGAAGTCACTGTTAAT	TTTGTTCGAAGGGCCCTCTAGA CTCGAG ACGTCTAAAACGACGTGT
HA-FUNDC1(S18A)	GAAAGTGATGATGACGCGTATGAAGTGT	CGCGTCATCATCACTTTCATGTTCTGGGGCGGT
HA-Δ1-10FUNDC1	AATTCGAGCTCATCGATGGTACCATGGA ACATGAAAGTGATGATGACTCC	TTAATTAAGATCTGCTAGCTCGAGTTAC GACGCAAGGCCCAAC
HA-Δ1-20FUNDC1	AATTCGAGCTCATCGATGGTA CCATGGTGTTGGACCTAACGGAATAT	TTAATTAAGATCTGCTAGCTCGAGTTAC GACGCAAGGCCCAAC
HA-Δ1-30FUNDC1	AATTCGAGCTCATCGATGGTACCATGC ACTGGTGGAATCGTTTGTTTGGC	TTAATTAAGATCTGCTAGCTCGAGT TACGACGCAAGGCCCAAC
HA-Δ1-40FUNDC1	AATTCGAGCTCATCGATGGTACCATG TCAGGACCAATTGTAGAAAAATAC	TTAATTAAGATCTGCTAGCTCGAGT TACGACGCAAGGCCCAAC
HA-Δ1-50FUNDC1	GAATTCGAGCTCATCGATGGTACCATG TCTGTAGCTACACAGATCGTGATGGGT	TTAATTAAGATCTGCTAGCTCGAGT TACGACGCAAGGCCCAAC
HA-FUNDC1(LIR)	GCAGAAGTGGCAGACCTAA CGGAATATGCAAGGC	TAGGTCTGCCACTTCTGCGGAGTCAT CATCACTTTCATGTTCTG
S-N240A	ACACAGGCGCATTCTCAGACGGCTTCTATCCGT	TGAGAATGCGCCTGTGTTGTACTGACAAGCCA
S-V443A	AGAGCAGGTCAGGGTTTCATTACGAACGTGAC	AAACCCTGACCTGCTCTGCCGTAGATTGAGTAGTCCA
S-N506A	GTTGTCAGCGGACGAGCAATCGT	GATTGCTCGTCCGCTGACAACAAACTGC
S-VE522AA	TGGTAGCGAACAGGCAGCAAACCAG	TTGCTGCCTGTTCGCTACCAGTTTCG

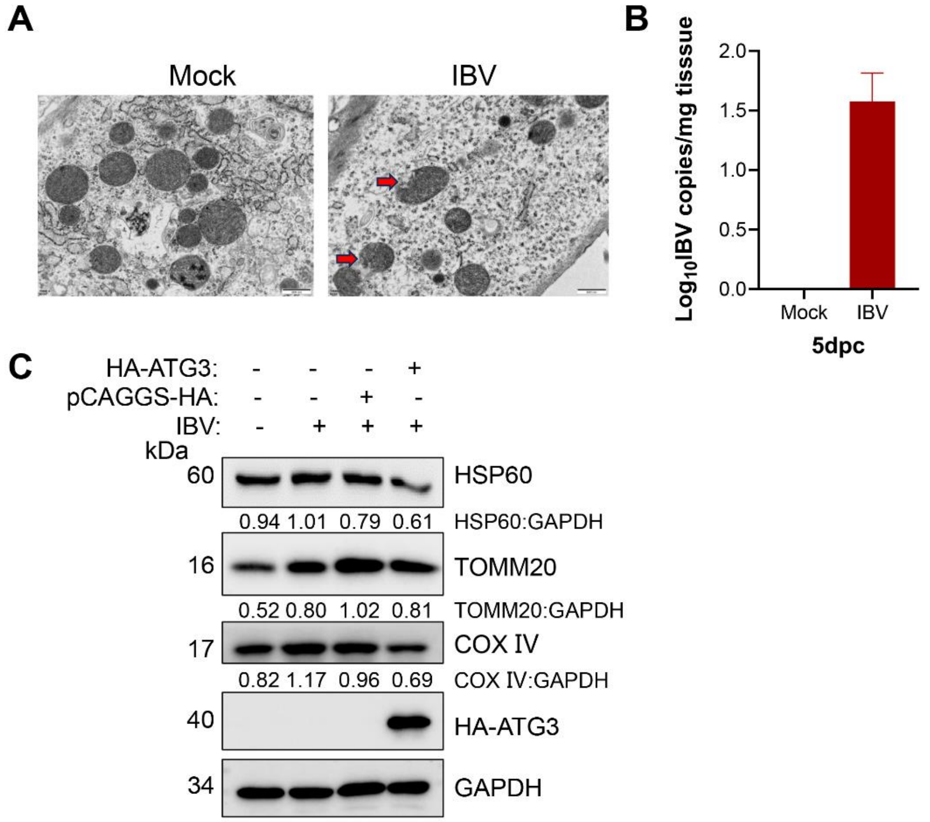

FIG S1 IBV infection inhibits mitochondrial autophagy. (A) CEK cells were infected with IBV for 24 h and examined for mitochondrial morphology by TEM. (B) 1-day-old SPF chickens were infected with IBV for 5 days, and viral loads in kidney tissues were determined by RT-qPCR. (C) CEK cells were transfected with HA-ATG3 for 12 h prior to IBV infection and harvested 24 h later for western blot detection of HSP60, TOMM20, and GAPDH.

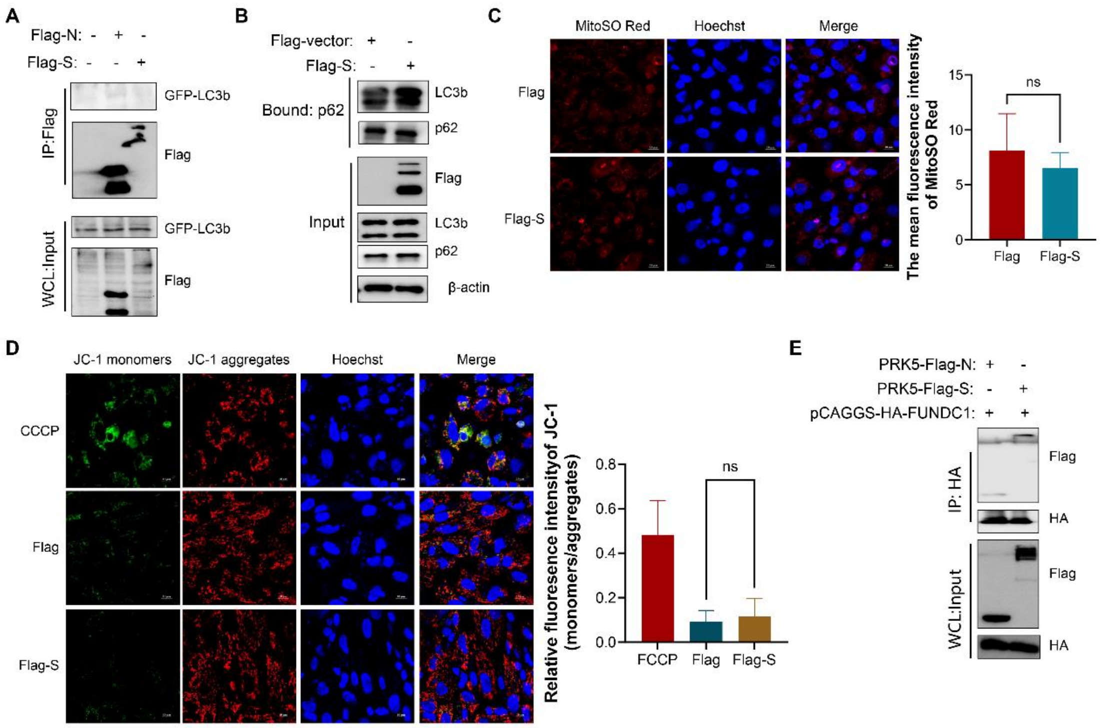

FIG S2 Interaction between the S protein and FUNDC1. (A) CEK cells were co-transfected with Flag-N, Flag-S, or empty vector together with GFP-LC3 for 24 h, and cell lysates were subjected to immunoprecipitation with an anti-Flag antibody. (B) CEK cells were transfected with Flag-S or empty vector for 24 h, followed by immunoprecipitation with an anti-p62 antibody. (C) CEK cells were transfected with the S protein. At 24 hours post-transfection, cells were incubated with MitoSOX™ Red working solution in a 37°C cell culture incubator for 60 minutes. Live cells were then observed under a laser scanning confocal microscope. Hoechst was used as a nuclear counterstain. (D) CEK cells were transfected with the S protein. At 24 hours post-transfection, JC-1 working solution was added and thoroughly mixed. Cells were incubated in a 37°C cell culture incubator for 20 minutes. CCCP served as a positive control and was applied at 5 μM for 20 minutes. Live cells were observed under a laser scanning confocal microscope. Hoechst was used as a nuclear counterstain. Images

were acquired and subsequently analyzed using ImageJ software. (E) CEK cells were co-transfected with Flag-N, Flag-S, and HA-FUNDC1 for 24 h, and cell lysates were subjected to immunoprecipitation with an anti-HA antibody. Data are mean ± SD from three independent experiments. Differences were considered significant at (*) $P < 0.05$, (**) $0.001 < P < 0.01$, (***) $P < 0.001$.

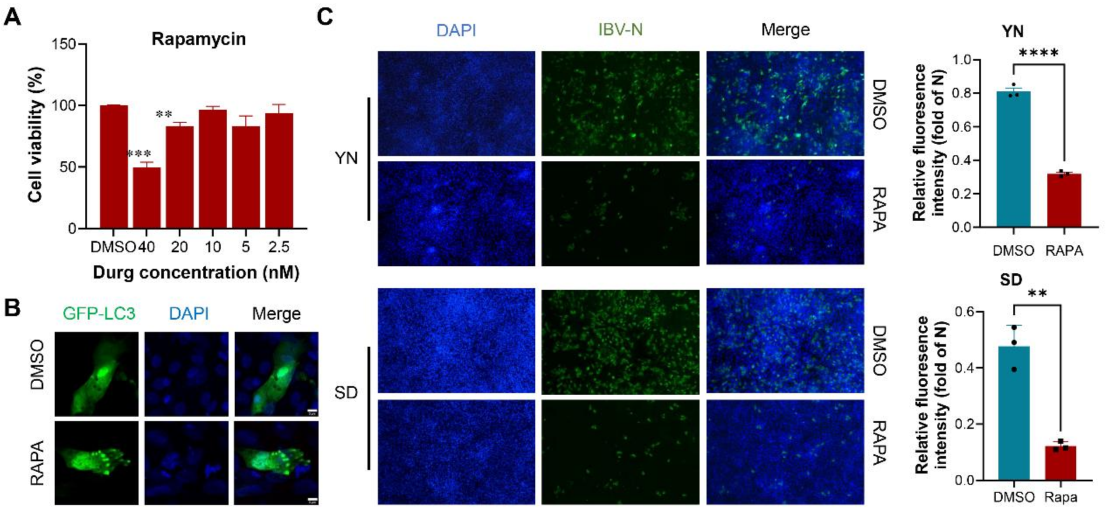

FIG S3 Rapamycin inhibits IBV replication. (A) CEK cells were treated with rapamycin (40 μM) at the indicated concentrations for 24 h, and cell viability was assessed using a CCK-8 assay. (B) CEK cells were transfected with GFP-LC3 for 6 h, followed by treatment with rapamycin (10 μM) for 24 h, and punctate GFP-LC3 structures indicative of autophagosome formation were visualized by confocal microscopy. (C) CEK cells were infected with IBV (YN or SD strains) for 2 h, followed by treatment with rapamycin (10 μM) or DMSO for 24 h, and viral N protein expression was examined by IFA. Results shown are representative of three independent experiments (mean ± SD) or of three independent experiments with similar results (one-way ANOVA in panels C). *P* value equal or lower to 0.05 was considered significant (**p* < 0.05, ***p* < 0.01, ****p* < 0.001). *P* value > 0.05 was considered statistically non-significant.

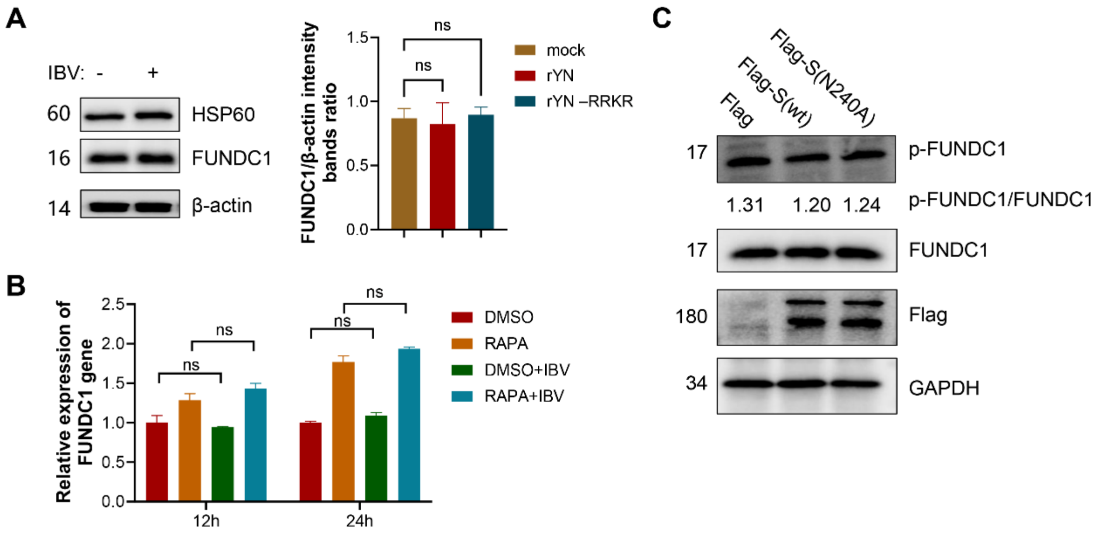

FIG S4 Determination of FUNDC1 levels in CEK. (A) CEK cells were infected with IBV for 24 h, and FUNDC1 levels were analyzed by Western blot. (B) CEK cells were treated with rapamycin for 24 h prior to infection with IBV (12 h or 24 h). Relative mRNA levels of FUNDC1 were measured by qPCR; β-actin served as the control. (C) CEK cells were transfected with Flag, Flag-S(WT), or Flag-S(N240A), respectively. Protein samples were collected 24 hours post-transfection, and the phosphorylation level of FUNDC1 was detected by western blot analysis.

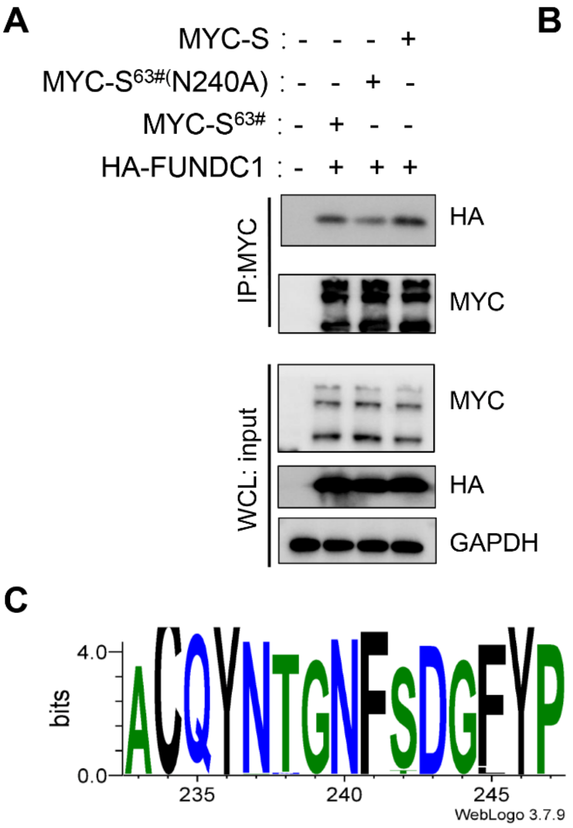

FIG S5 (A) DF-1 cells were co-transfected with HA-FUNDC1 and either wild-type or mutant MYC-S plasmids. Cell lysates were collected 24 hours post-transfection, and the interaction between HA and MYC was assessed by co-immunoprecipitation (co-IP) using anti-MYC magnetic beads. (B) DF-1 cells were co-transfected with HA-FUNDC1 and MYC-$S^{63\#}$ plasmids. Cell lysates were harvested 24 hours post-transfection, and the interaction between HA and MYC was examined by co-IP using anti-HA magnetic beads. (C) Schematic representation of the amino acid sequence spanning residues 233-247 of the IBV S protein.

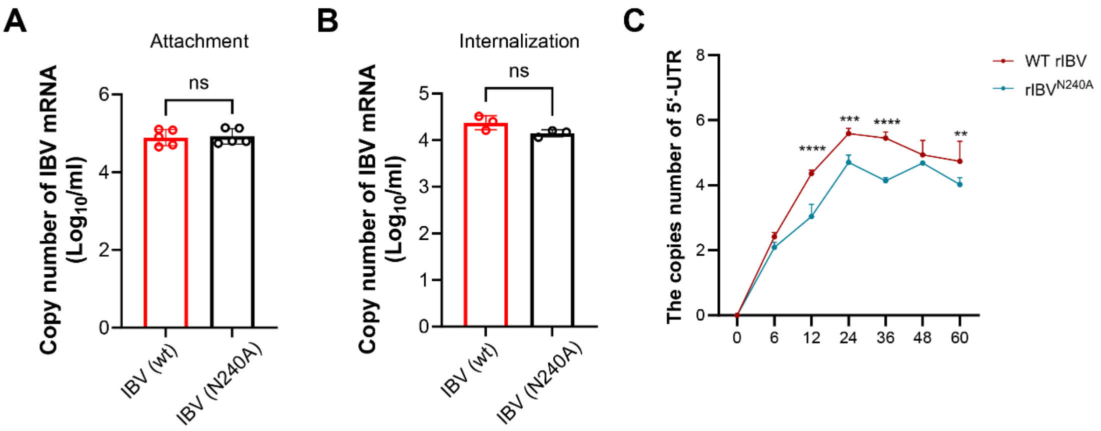

FIG S6 Impact of IBV (WT) versus IBV (N240A) mutation on viral entry. (A) CEK cells were infected with IBV at an MOI of 5 and incubated at 4°C for 1 hour. For the virus attachment assay, cells were washed three times with cold PBS (4°C) to remove unbound viral particles and then harvested for viral RNA extraction. Viral RNA levels were measured by RT-PCR. (B) For the internalization assay, infected cells as described above were further incubated with pre-warmed DMEM for 1 hour at 37°C. The cells were then treated with 1 mg/mL pronase in cold PBS to remove attached but non-internalized viral particles. After three washes, cells were lysed with TRIzol reagent to extract total cellular RNA, and viral RNA was quantified by RT-PCR. (C) One-step growth curve analysis (MOI = 5). Following infection of CEK cells for 1 hour, the cells were washed three times with PBS and replenished with fresh medium. Samples were collected at 6, 12, 24, 36, 48, and 60 hpi for viral copy number quantification.

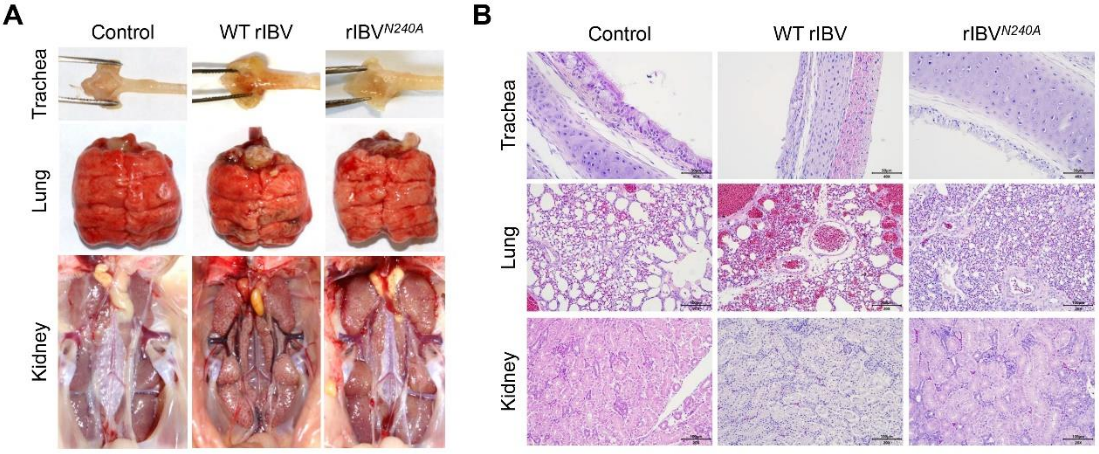

FIG S7 Pathological analysis of the pathogenicity of WT rIBV and rIBV ^{N240A} in SPF chickens. (A) Gross lesions of 1-day-old SPF chickens infected with WT rIBV and rIBV ^{N240A} at 5 days post-infection. (B) Histopathological analysis of different tissues from 1-day-old SPF chickens infected with WT rIBV and rIBV ^{N240A} at 5 days post-infection. Tissues were stained with hematoxylin and eosin (H&E) (Magnification, ×200/×400).

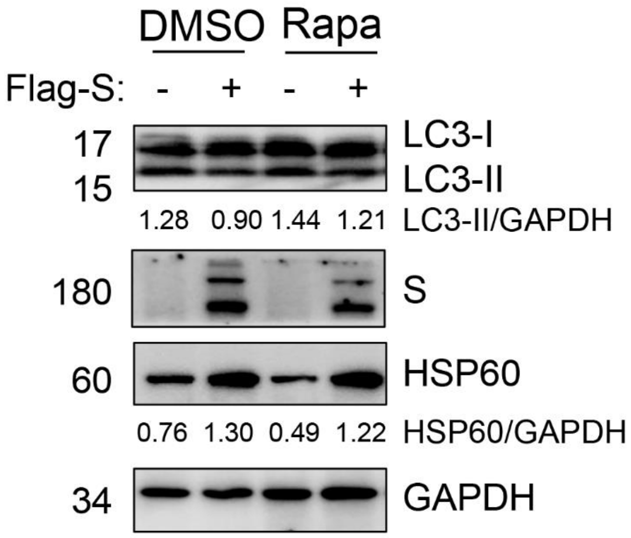

FIG S8 CEK cells were transfected with either an empty Flag vector or Flag-S. 6 hours post-transfection, the cells were treated with either DMSO or rapamycin for 24 hours. Protein samples were then collected, and the levels of LC3 and HSP60 were detected by western blot analysis.
